# Supplementary material for: Characteristics and management of adolescents attending the ED with fever: a prospective multicentre study
Source: BMJ Open. 2022 Jan 19;12(1):e053451. doi: 10.1136/bmjopen-2021-053451 (PMC8772429; doi:10.1136/bmjopen-2021-053451)
Supplement: Supplementary data [file bmjopen-2021-053451supp002.pdf]

Appendix 2: Participating EDs

Hospital	Country, city	Hospital type	Total annual paediatric ED visits	Total period of inclusion	Period of inclusion per month	Number of patients included
Medizinische Universität Graz	Austria, Graz	University	10,000-30,000	1-1-2017 – 31-12-2018	10 days	2243
Dr. von Hauner Children's Hospital	Germany, Munich	Teaching	10,000-30,000	1-1-2017 – 31-12-2018	1 week	1175
P. and A. Kyriakou Children's Hospital	Greece, Athens	University	>30,000	1-1-2017 – 1-5-2018	1-2 weeks	4549
Children clinical university hospital	Latvia, Riga	Teaching	>30,000	1-1-2017 – 31-12-2018	All	9000
Univerzitetni Klinični Center	Slovenia, Ljubljana	University	<10,000	1-1-2017 – 31-12-2018	All	3659
Hospital Clínico Universitario	Spain, Santiago de Compostela	University	>30,000	1-1-2017 – 1-5-2018	1-2 weeks	3877
Erasmus MC-Sophia Children's Hospital	The Netherlands, Rotterdam	University	<10,000	1-1-2017 – 1-4-2018	All	1681
RadboudUMC	The Netherlands, Nijmegen	University	<10,000	1-1-2017 – 1-4-2018	All	676
Canisius Wilhelmina Ziekenhuis	The Netherlands, Nijmegen	Teaching	<10,000	1-1-2017 – 31-12-2018	2 weeks	415
Alder Hey Children's Hospital	United Kingdom, Liverpool	Teaching	>30,000	1-1-2017 – 31-12-2018	1 week	1624
St. Mary's Hospital	United Kingdom, London	University	10,000-30,000	1-1-2017 – 31-12-2018	All	5714
Great North Children's Hospital	United Kingdom, Newcastle upon Tyne	University	>30,000	1-4-2017 – 1-4-2018	2 weeks	3870

Borensztajn D, *et al. BMJ Open* 2022; 12:e053451. doi: 10.1136/bmjopen-2021-053451
