# Supplementary material for: Characteristics and management of adolescents attending the ED with fever: a prospective multicentre study
Source: BMJ Open. 2022 Jan 19;12(1):e053451. doi: 10.1136/bmjopen-2021-053451 (PMC8772429; doi:10.1136/bmjopen-2021-053451)
Supplement: Supplementary data [file bmjopen-2021-053451supp003.pdf]

Appendix 3. Immediate life-saving interventions

1. Airway and breathing support, including intubation or emergent noninvasive positive pressure ventilation.
2. Electrical therapy, including defibrillation, emergent cardioversion, or external pacing.
3. Procedures, including chest needle decompression, pericardiocentesis, or open thoracotomy.
4. Hemodynamic support, including significant intravenous fluid resuscitation in the setting of hypotension, blood administration, or control of major bleeding.
5. Emergency medications, including naloxone, dextrose, atropine, adenosine, epinephrine, or vasopressors

Borensztajn D*, et al. BMJ Open* 2022; 12:e053451. doi: 10.1136/bmjopen-2021-053451
