# Supplementary figures and images for: Characteristics and management of adolescents attending the ED with fever: a prospective multicentre study
Source: BMJ Open. 2022 Jan 19;12(1):e053451. doi: 10.1136/bmjopen-2021-053451 (PMC8772429; doi:10.1136/bmjopen-2021-053451)

## Presumed cause of infection: categorisation based on clinical data

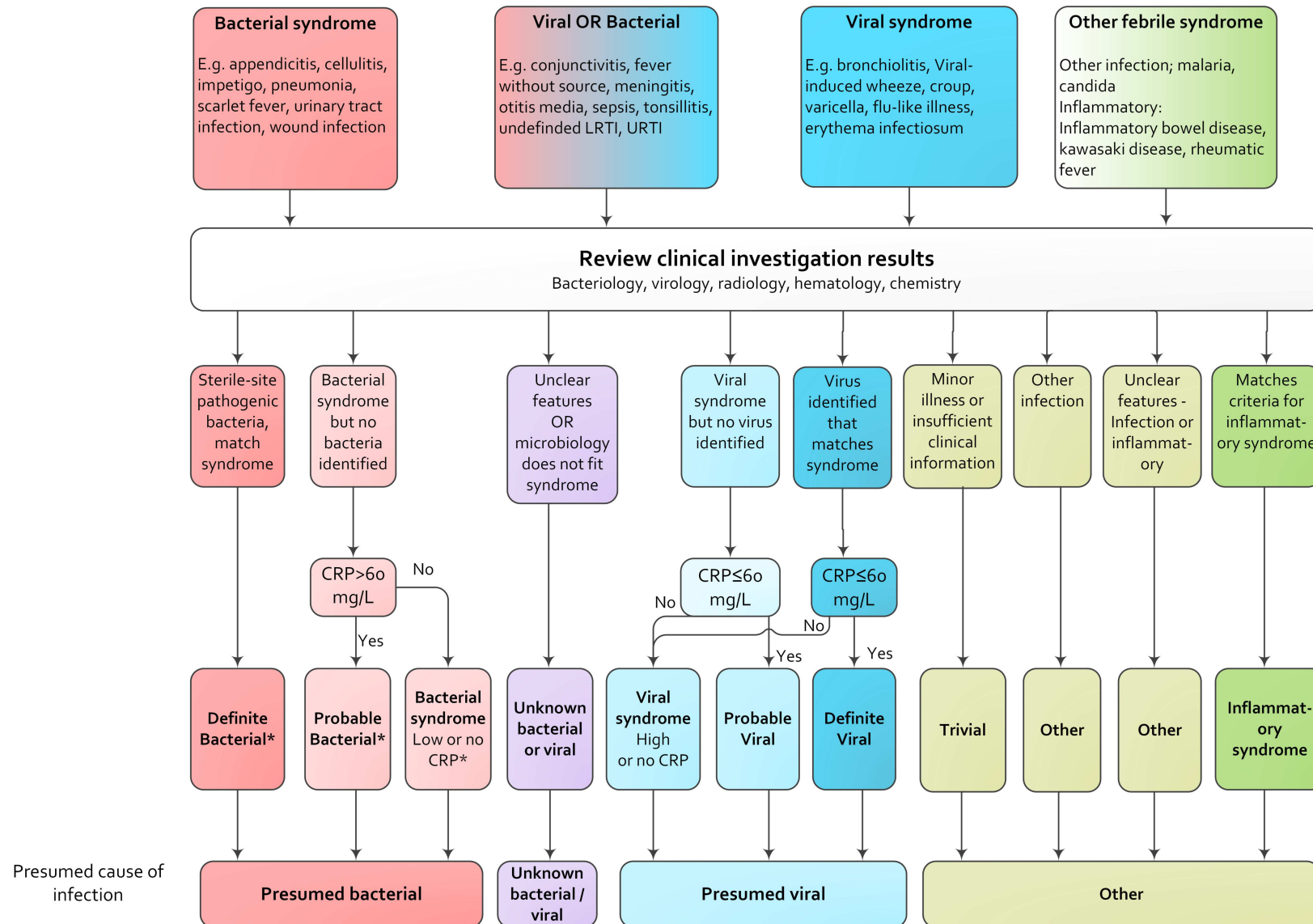

Supplement: Supplementary data [file bmjopen-2021-053451supp004.pdf]
