# Supplementary material for: Characteristics and management of adolescents attending the ED with fever: a prospective multicentre study
Source: BMJ Open. 2022 Jan 19;12(1):e053451. doi: 10.1136/bmjopen-2021-053451 (PMC8772429; doi:10.1136/bmjopen-2021-053451)
Supplement: Supplementary data [file bmjopen-2021-053451supp005.pdf]

Appendix 5: Comorbidity.

	**Children<br>3 months- 12 years<br>N = 34,843 N (%)**	**Children<br>> 12 years<br>N = 2,577 N (%)**
**No comorbidity**	**28,881 (83·7)**	**1,833 (71·5)**
**Non-complex comorbidity**	**4,302 (12·5)**	**489 (19·1)**
**Complex comorbidity**	**1,332 (3·9)**	**241 (9·4)**
**Type of comorbidity**		
**Neurological & psychomotor delay**	**1,604 (28·5)**	**298 (40·8)**
**Pulmonary**	**1,224 (21·7)**	**182 (24·9)**
**Prematurity**	**945 (16·8)**	**20 (2·7)**
**Urological/nephrological**	**634 (11·3)**	**57 (7·8)**
**Malignancy & immunodeficiency**	**583 (10·4)**	**162 (22·2)**
**Cardiac**	**557 (9·9)**	**58 (7·9)**
**Gastro-intestinal**	**422 (7·5)**	**81 (11·1)**
**Musculoskeletal**	**171 (3·0)**	**55 (7·5)**
**Metabolic**	**165 (2·9)**	**53 (7·3)**
**Endocrine**	**63 (1·1)**	**27 (3·7)**
**Other comorbidity**	**729 (12·9)**	**83 (11·3)**

Type of comorbidity displayed as percentage of children with comorbidity.
