# Supplementary material for: Characteristics and management of adolescents attending the ED with fever: a prospective multicentre study
Source: BMJ Open. 2022 Jan 19;12(1):e053451. doi: 10.1136/bmjopen-2021-053451 (PMC8772429; doi:10.1136/bmjopen-2021-053451)
Supplement: Supplementary data [file bmjopen-2021-053451supp006.pdf]

Appendix 6: Differences in patient characteristics between young children and adolescents with a final diagnosis of SBI. [a]

	OR	Lower limit	Upper limit
General patient characteristics			
Triage urgency high	1.0	0.8	1.3
Referral by EMS	1.0	1.0	1.1
Comorbidity	1.7	1.4	2.2
Vital signs			
Tachycardia	0.9	0.7	1.2
Tachypnoea	0.5	0.3	0.6
Prolonged capillary refill	1.1	0.6	2.2

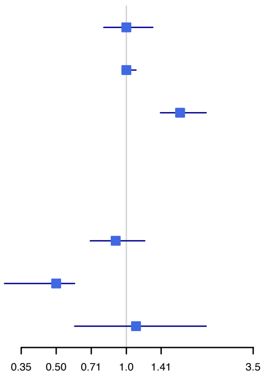

	OR	Lower limit	Upper limit
NICE fever guideline alarming signs			
Ill appearance	1.4	1.1	1.8
Non−blanching rash	0.8	0.3	2.1
Blanching rash	0.7	0.4	1.1
Increased work of breathing	0.4	0.3	0.7
Neurological signs	1.1	0.3	3.1
NICE sepsis guideline high risk criteria			
1 or more	0.4	0.3	0.5
2 or more	0.3	0.2	0.4
3 or more	0.2	0.1	0.3

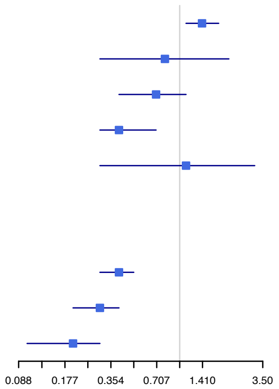

[a] Younger children used as reference. Adjusted for hospital, sex, duration of fever, previous medical care, time of arrival and comorbidity. * According to APLS cut-off values by age.
