# Supplementary material for: Characteristics and management of adolescents attending the ED with fever: a prospective multicentre study
Source: BMJ Open. 2022 Jan 19;12(1):e053451. doi: 10.1136/bmjopen-2021-053451 (PMC8772429; doi:10.1136/bmjopen-2021-053451)
Supplement: Author's manuscript [file bmjopen-2021-053451.draft_revisions.pdf]

BMJ Open is committed to open peer review. As part of this commitment we make the peer review history of every article we publish publicly available.

When an article is published we post the peer reviewers' comments and the authors' responses online. We also post the versions of the paper that were used during peer review. These are the versions that the peer review comments apply to.

The versions of the paper that follow are the versions that were submitted during the peer review process. They are not the versions of record or the final published versions. They should not be cited or distributed as the published version of this manuscript.

BMJ Open is an open access journal and the full, final, typeset and author-corrected version of record of the manuscript is available on our site with no access controls, subscription charges or pay-per-view fees (http://bmjopen.bmj.com).

If you have any questions on BMJ Open's open peer review process please email info.bmjopen@bmj.com

BMJ Open

The adolescent paradox at the ED: teenagers visiting the ED have an increased risk of serious bacterial infections.

Journal:	*BMJ Open*
Manuscript ID	bmjopen-2021-053451
Article Type:	Original research
Date Submitted by the Author:	13-May-2021

Complete List of Authors:	Borensztajn, Dorine; Erasmus MC Sophia Children Hospital, Department of Pediatrics<br>Hagedoorn, Nienke; Erasmus MC Sophia Children Hospital, Department of Pediatrics<br>Carrol, Enitan; University of Liverpool, Institute of Infection and Global Health<br>von Both, Ulrich; Munich University Hospital Dr von Hauner Children's Hospital<br>Dewez, Juan ; London School of Hygiene and Tropical Medicine, Clinical Research Department<br>Emonts, Marieke; Great North Children's Hospital, Paediatric Immunology, Infectious Diseases & Allergy, Newcastle upon Tyne Hospitals NHS Foundation Trust<br>van der Flier, Michiel; Radboudumc, Pediatric Infectious Diseases and Immunology<br>de Groot, Ronald; Radboudumc, Pediatric Infectious Diseases and Immunology<br>Herberg, Jethro; Imperial College London, Paediatrics<br>Kohlmaier, Benno; Medical University of Graz, Department of General Paediatrics<br>Levin, M<br>Lim, Emma; Great North Children's Hospital<br>Maconochie, Ian; Imperial College London, Paediatric Emergency Department<br>Martinon Torres, Frederico; University Hospital of Santiago de Compostela, Pediatrics<br>Nijman, R; Imperial College Healthcare NHS Trust, Department of Paediatrics<br>Pokorn, Marko; University of Ljubljana, Department of Infectious Diseases<br>Rivero-Calle, Irene; University Hospital of Santiago de Compostela, Pediatrics<br>Tsolia, Maria; National and Kapodistrian University of Athens, Department of Paediatric Infectious Diseases, University of Athens<br>Vermont , Clementien ; Erasmus MC Sophia Children Hospital, Department of paediatric infectious diseases and immunology<br>Zavadska, Dace; Riga Stradins University, Department of Pediatrics<br>Zenz, Werner; Medical University of Graz, Department of General Paediatrics<br>Zachariasse, Joany; Erasmus MC Sophia,<br>moll, Henriette; Erasmus MC Sophia Children Hospital, Department of

	Pediatrics
Keywords:	PAEDIATRICS, Paediatric infectious disease & immunisation < PAEDIATRICS, Paediatric A&E and ambulatory care < PAEDIATRICS

SCHOLARONE™
Manuscripts

I, the Submitting Author has the right to grant and does grant on behalf of all authors of the Work (as defined in the below author licence), an exclusive licence and/or a non-exclusive licence for contributions from authors who are: i) UK Crown employees; ii) where BMJ has agreed a CC-BY licence shall apply, and/or iii) in accordance with the terms applicable for US Federal Government officers or employees acting as part of their official duties; on a worldwide, perpetual, irrevocable, royalty-free basis to BMJ Publishing Group Ltd ("BMJ") its licensees and where the relevant Journal is co-owned by BMJ to the co-owners of the Journal, to publish the Work in this journal and any other BMJ products and to exploit all rights, as set out in our licence.

The Submitting Author accepts and understands that any supply made under these terms is made by BMJ to the Submitting Author unless you are acting as an employee on behalf of your employer or a postgraduate student of an affiliated institution which is paying any applicable article publishing charge ("APC") for Open Access articles. Where the Submitting Author wishes to make the Work available on an Open Access basis (and intends to pay the relevant APC), the terms of reuse of such Open Access shall be governed by a Creative Commons licence – details of these licences and which Creative Commons licence will apply to this Work are set out in our licence referred to above.

Other than as permitted in any relevant BMJ Author's Self Archiving Policies, I confirm this Work has not been accepted for publication elsewhere, is not being considered for publication elsewhere and does not duplicate material already published. I confirm all authors consent to publication of this Work and authorise the granting of this licence.

For peer review only

The adolescent paradox at the ED: teenagers visiting the ED have an increased risk of serious bacterial infections.

Dorine M. Borensztajn[1], Nienke N. Hagedoorn[1], Enitan D. Carrol[2,3,4], Ulrich von Both[,5,6], Juan Emmanuel Dewez[7], Marieke Emonts[,8,9,10], Michiel van der Flier[11,12], Ronald de Groot[11], Jethro Herberg[13], Benno Kohlmaier[14], Michael Levin[13,] Emma Lim[8], Ian K. Maconochie[13], Federico Martinon-Torres[15], Ruud G. Nijman[13], Marko Pokorn[16], Irene Rivero-Calle[15], Maria Tsolia[17], Clementien Vermont[18], Dace Zavadska[19], Werner Zenz[14], Joany M. Zachariasse[1], Henriette A. Moll[1]

On behalf of PERFORM consortium: Personalised Risk assessment in febrile children to optimise Real-life Management across the European Union.*

Corresponding author:

Dorine Borensztajn

Department of General Paediatrics, Erasmus MC – Sophia Children's Hospital

P.O. Box 2060, 3000 CB, Rotterdam, the Netherlands

Telephone: +31 10 703 61 96

E-mail: d.borensztajn@erasmusmc.nl

Author affiliations:

1. Erasmus MC-Sophia Children's Hospital, Department of General Paediatrics, Rotterdam, the Netherlands.

For peer review only

2. University of Liverpool, Institute of Infection, Veterinary and Ecological Sciences, Liverpool, United Kingdom.
3. Alder Hey Children's NHS Foundation Trust, Department of Infectious Diseases, Liverpool, UK
4. Liverpool Health Partners, Liverpool, UK
5. Dr. von Hauner Children's Hospital, University hospital, Ludwig-Maximilians-University (LMU), Division of Paediatric Infectious Diseases, München, Germany.
6. German Centre for Infection Research, DZIF, partner site Munich, Germany
7. London School of Hygiene and Tropical Medicine, Faculty of Tropical and Infectious Disease, London, United Kingdom.
8. Great North Children's Hospital, Paediatric Immunology, Infectious Diseases & Allergy, Newcastle upon Tyne Hospitals NHS Foundation Trust, Newcastle upon Tyne, United Kingdom.
9. Translational and Clinical Research Institute, Newcastle University, Newcastle upon Tyne, United Kingdom
10. NIHR Newcastle Biomedical Research Centre based at Newcastle upon Tyne Hospitals NHS Trust and Newcastle University, Newcastle upon Tyne, United Kingdom
11. Stichting Katholieke Universiteit, Radboudumc Nijmegen, The Netherlands.
12. Wilhelmina Children's Hospital, University Medical Centre Utrecht, Department of Paediatric Infectious Diseases and Immunology, Utrecht, The Netherlands.
13. Imperial College of Science, Technology and Medicine, Section of Paediatric Infectious Diseases, London, United Kingdom.
14. Medical University of Graz, Department of General Paediatrics, Graz, Austria.

For peer review only

15. Hospital Clínico Universitario de Santiago de Compostela, Genetics, Vaccines, Infections and Pediatrics Research group (GENVIP), Santiago de Compostela, Spain
16. University Medical Centre Ljubljana, Univerzitetni Klinični Center, Department of Infectious Diseases, Ljubljana, Slovenia.
17. National and Kapodistrian University of Athens, Second Department of Paediatrics, P. and A. Kyriakou Children's Hospital, Second Department of Paediatrics, Athens, Greece.
18. Erasmus MC-Sophia Children's Hospital, Department of Pediatric infectious diseases & immunology, Rotterdam, the Netherlands.
19. Rīgas Stradiņa universitāte, Children clinical university hospital, Department of Pediatrics, Riga, Latvia

*Membership of the PERFORM Consortium is provided in appendix 1.

For peer review only

Abbreviations

aOR – adjusted odds ratio

AUC – Area under the Curve

ED – Emergency Department

EMS – Emergency Medical Services

IBI – Invasive bacterial infection

ILSI – Immediate life-saving interventions

MOFICHE – Management and Outcome of Fever in children in Europe

NICE - National Institute for Health and Care Excellence

OR – odds ratio

For peer review only - http://bmjopen.bmj.com/site/about/guidelines.xhtml

PERFORM - Personalized Risk assessment in Febrile illness to Optimize Real-life Management across the European Union

PEWS - Paediatric Early Warning Score

PICU – Paediatric Intensive Care Unit

SBI – Serious bacterial infection

Word count: 2516

Funding:

This project has received funding from the European Union's Horizon 2020 research and innovation programme under grant agreement No. 668303

The Research was supported by the National Institute for Health Research Biomedical Research Centre based at Imperial College (JH, ML) and at Newcastle Hospitals NHS Foundation Trust and Newcastle University (EL, ME), grant number not applicable.

The views expressed are those of the author(s) and not necessarily those of the NHS, the NIHR or the Department of Health.

Contributors:

All authors contributed to the design of the study, DB and NH collected the data. DB and NH verified and analysed the data. DB, NH and HM interpreted the data. DB, NH and HM drafted the manuscript. All authors critically evaluated and revised the manuscript. The corresponding author attests that all listed authors meet authorship criteria and that no others meeting the criteria have been omitted. This publication is the work of the authors who will serve as guarantors for the contents of this paper.

Competing interest:

The authors declare they have no potential conflicts of interest to disclose.

For peer review only - http://bmjopen.bmj.com/site/about/guidelines.xhtml

For peer review only

Data:

An anonymized data set necessary to replicate our study findings will be provided as a public repository with a DOI in case of acceptance of the manuscript.

For peer review only

For peer review only - http://bmjopen.bmj.com/site/about/guidelines.xhtml

Abstract

Objective

Most studies regarding febrile children have focused on infants and young children with serious bacterial infections (SBI). Although population studies have described an increased risk of sepsis in adolescents, little is known about febrile adolescents attending the Emergency Department (ED). We aimed to describe patient characteristics and management of febrile adolescents attending the ED.

Design and setting

The MOFICHE/PERFORM, a prospective multicentre study, took place at 12 European EDs. Descriptive and multivariable regression analysis were performed, comparing febrile adolescents (12-18 years) to younger children in terms of patient characteristics, markers of disease severity (vital signs, clinical alarming signs), management (diagnostic tests, therapy, admission) and diagnosis (focus, viral/bacterial infection).

Results

37,420 encounters were included, of which 2,577 (6,9%) were adolescents.

Adolescents were more often triaged as high-urgent (38·9% versus 34·5%) and described as ill-appearing (23·1% versus 15·6%) than younger children.

Increased work of breathing and a non-blanching rash were present less often in adolescents while neurological signs were present more often (1% versus 0%). C-reactive protein was performed more frequently in adolescents and was abnormal more often (aOR 1·7, 95%CI 1·5-1·9). Adolescents were more often diagnosed with SBI (OR 1·8, 95%CI 1·6-2·0) and sepsis/meningitis (OR 2·3, 95%CI 1·1-5·0), more frequently were admitted (aOR 1·3, 95% CI 1·2-1·4) and treated with intravenous antibiotics (aOR 1·7, 95% CI 1·5-2·0).

For peer review only - http://bmjopen.bmj.com/site/about/guidelines.xhtml

Conclusions

Although younger children presented to the ED more frequently, adolescents were more often diagnosed with SBI and sepsis/meningitis. Our data emphasize the importance of awareness for severe infections in adolescents.

Keywords

Adolescent health, epidemiology.

For peer review only

Article summary (strengths and limitations of this study)

- Our study provides detailed data on a large number of adolescents attending the ED with fever
- Our data show that although accounting for a relatively small fraction of all ED visits for febrile children, adolescents have an increased risk of serious bacterial infections
- Febrile adolescents are hospitalized more often, are treated with intravenous antibiotics more often and more often required immediate life-saving interventions
- Adolescents with serious bacterial infections present differently than younger children and more research is needed to be able to provide detailed guidelines for this age group

INTRODUCTION

The adolescent age is often described as a health paradox because on the one hand it is a time of enhanced physical and mental capabilities, yet the overall mortality/morbidity rates increase significantly, often due to risk taking behaviour such as substance abuse or injuries.[1]

Although this health paradox has received considerable attention in the international literature, there is a knowledge gap regarding infectious problems in adolescents, as most

For peer review only - http://bmjopen.bmj.com/site/about/guidelines.xhtml

studies on adolescents have focused on topics typically associated with adolescence, such as violence or mental health,[2-4] while most studies on infections have focused on younger children.[5]

Case series as well as population studies have shown adolescents to have an increased risk of sepsis in comparison to younger children,[6,7] and several studies showed adolescents with sepsis to have an increased mortality rate.[6-11]

One possible explanation of this increased case-fatality rate might be the atypical presentation of adolescents with sepsis, such as gastro-intestinal complaints.[9,12]

The increased incidence of sepsis and the high case-fatality rates emphasize the importance of awareness for severe infections in adolescents.

Despite this, little is known on the presentation, management and diagnosis of febrile adolescents presenting to the ED.

Our aim was to assess the presentation, management and diagnosis of febrile adolescents attending the ED and explore differences between adolescents and younger children.

METHODS

Study design

This study is part of the MOFICHE study (Management and Outcome of Febrile children in Europe), which is embedded in the PERFORM study (Personalized Risk assessment in Febrile illness to Optimise Real-life Management across the European Union).[13] MOFICHE is an observational multicentre study that evaluates the management and outcome of febrile children in Europe using routinely collected data.[14] The study was approved by the ethical committees of all the participating hospitals and no informed consent was needed for this

For peer review only

For peer review only - http://bmjopen.bmj.com/site/about/guidelines.xhtml

study. Austria (Ethikkommission Medizinische Universitat Graz, ID:28-518ex15/16), Germany (Ethikkommission Bei Der LMU München, ID:699-16), Greece (Ethics committee, ID:9683/18.07.2016), Latvia (Centrala medicinas etikas komiteja, ID:14.07.201.6.No. Il16-07-14), Slovenia (Republic of Slovenia National Medical Ethics Committee, ID:0120-483/2016-3), Spain (Comité Autonómico de Ética de la Investigación de Galicia, ID:2016/331), The Netherlands (Commissie Mensgebonden onderzoek, ID:NL58103.091.16), United Kingdom (Ethics Committee, ID:16/LO/1684, IRAS application no. 209035, Confidentiality advisory group reference: 16/CAG/0136).

In all the participating UK settings, an additional opt-out mechanism was in place.

Patient and public involvement

Patients were not involved in the design of the study.

Study population and setting

Twelve EDs from eight different countries (Austria, Germany, Greece, Latvia, the Netherlands (n=3), Spain, Slovenia, and the United Kingdom (n=3)) participated in the study. Participating hospitals were either tertiary university hospitals or large teaching hospitals (appendix 2). Data were collected for at least one year (January 2017-April 2018).

For this analysis, inclusion criteria were children aged 3 months-18 years presenting to the ED with fever (temperature >=38·0° C) or a history of fever in the previous 72 hours.

Data collection

Data were obtained from patient records and entered into an electronic case report form. Data included general patient characteristics (age, sex, comorbidity, previous medical care,

For peer review only - http://bmjopen.bmj.com/site/about/guidelines.xhtml

arrival time, referral (self, primary care physician, Emergency Medical Services (EMS) or other), triage urgency, vital signs, presence of "red traffic light" alarming signs from the National Institute for Health and Care Excellence (NICE) fever guideline,[15] high risk criteria from the NICE sepsis guideline[15] (table 1) and management at the ED. The NICE alarming signs include level of consciousness, ill-appearance, increased work of breathing, age <3 months, non-blanching rash, meningeal signs, status epilepticus and focal neurological signs. High risk criteria from the NICE Sepsis guideline overlap with NICE alarming signs but differ by age-group and include abnormal behaviour, decreased consciousness, low oxygen saturation, abnormal heart rate, respiratory rate or blood pressure, hypothermia, age <3 months, diminished urine output, cyanosis or a non-blanching rash. An overview of the collected alarming signs is provided in table 2.

Data collection ranged from one week per month to the entire month, depending on the number of ED visits per hospital (appendix 2).

Definitions

Adolescents were defined as children aged 12-18 years; younger children were defined as children aged 3 months-12 years.

Previous medical care was defined as medical care for the same complaint in the last 5 days at any facility. Comorbidity was defined as a chronic underlying condition that is expected to last at least 1 year.[16]

Vital signs were classified as abnormal according to APLS reference ranges.[17]

Simplified Paediatric Early Warning Scores (PEWS) were calculated based on the PEWS developed by Parshuram et al. (vital signs, capillary refill time, work of breathing and oxygen therapy, combined into a score).[18,19] Blood pressure was excluded from the PEWS as it was

For peer review only - http://bmjopen.bmj.com/site/about/guidelines.xhtml

not routinely performed in our study. A previous study showed that a simplified PEWS without blood pressure showed similar performance in predicting PICU admission in comparison to the original PEWS.[19]

Triage categories were combined into “low urgency” (non-urgent and standard) and “high urgency” (urgent, very urgent and immediate).

Immediate life-saving interventions[20] (ILSI) were categorized into the following categories: airway/breathing support, electrical therapy, emergency procedures, hemodynamic support and emergency medications (appendix 3).

Focus of infection was categorized as: upper respiratory, lower respiratory, gastro-intestinal/surgical abdomen, urinary, skin, musculoskeletal, sepsis, meningitis/central nervous system (CNS), flu-like illness, childhood exanthemas, inflammatory, undifferentiated fever or other.[14]

The consortium developed a consensus-based flowchart[14,21,22] to classify the presumed cause of infection for each visit (appendix 4), depending on clinical signs, CRP and microbiological tests (bacterial cultures, viral or bacterial PCR), into “definite or probable bacterial”, “definite or probable viral”, “unknown” or “other”.

Serious bacterial infection (SBI) was defined as ‘definite/probable bacterial’ with a focus from the gastro-intestinal, lower respiratory, urinary or musculoskeletal tract, CNS or sepsis. Sepsis/meningitis was defined as ‘definite/probable bacterial’ with a focus from the CNS or sepsis.

For peer review only - http://bmjopen.bmj.com/site/about/guidelines.xhtml

Data quality and missing data

Data quality and completeness was improved and standardized by using a digital training module for physicians who assess febrile children at the ED, including clarification of the NICE alarming signs. Data were entered into the patient's record as part of routine care by the treating physician and nurse and were then manually extracted from these records and entered into an electronic case report form by trained research team members.

Missing determinants such as vital signs were handled by multiple imputation (table 1). Imputation was performed by using the MICE package in R, version 3.4. SPSS version 25 was used for the analysis of the data.

Data analysis

We performed descriptive analyses for general patient characteristics, vital signs, PEWS, NICE alarming signs, management (diagnostic tests, intravenous antibiotics, oxygen therapy, ILSI, disposition (discharge, hospital admission, PICU admission) and diagnosis (focus of infection, viral or bacterial disease). Characteristics of adolescents and younger children were compared using chi-squared-tests and Mann-Whitney tests. Results were deemed significant with a p-value <0·05.

We analysed differences in management, disposition and presumed cause of infection by multivariable logistic regression adjusted for general patient characteristics (setting/ED, sex, fever duration, previous medical care, arrival time and comorbidity). We did not adjust for disease severity as our aim was to describe differences in disease severity between young children and adolescents. Subgroup analyses were performed for children and adolescents diagnosed with SBI and for children without comorbidity.

For peer review only

For peer review only - http://bmjopen.bmj.com/site/about/guidelines.xhtml

RESULTS

Patient characteristics

37,420 ED encounters were included, of which 2,577 (6·9%) were adolescents (table 1).

Adolescents were less often self-referred (49·4% versus 57·8%) and more often presented by EMS than younger children (17·3% versus 14·8%, p< 0·001).

Adolescents more often had comorbidity (28·5% versus 16·4%, p<0·001, table 1, appendix 5).

Presenting signs and symptoms

Adolescents were more often triaged as high urgent. Tachycardia was present more often (29·6% versus 24·5%, p<0·001) while tachypnoea, increased work of breathing and low oxygen saturation were present less often.

Adolescents more often had a PEWS score of 6 or higher (6·2% versus 4·5%, p<0·001).

Non-blanching rashes were present less often in adolescents while ill-appearance, meningeal signs and focal neurological signs were present more often (table 1).

In a sub-analysis of children without comorbidity, results were similar, except triage urgency, PEWS and focal neurological signs, which were then similar in both groups.

Management

After adjusting for general patient characteristics, we found that diagnostic tests were such as CRP were performed more often in adolescents (aOR 1·9, 95% CI 1·7-2·0) and CRP more often reached levels >60 mg/l (aOR 1·7, 95% CI 1·5-1·9).

For peer review only

Hospital admission (aOR 1·3, 95% CI 1·2-1·4), intravenous antibiotics (aOR 1·7, 95% CI 1·5-1·9) and ILSI (aOR 1·5, 95% 1·2-2·0) were more common in adolescents while PICU admission was similar in both age-groups (aOR 1·1, 95% CI 0·6-1·9, figure 1).

Sub-analysis in children without comorbidity showed similar results, except for ILSI, which was similar in both groups (figure 3).

Focus and presumed cause of infection

Upper respiratory tract infection was the most common focus in both age-groups, although this was less common in adolescents than in younger children (41·8% versus 53·9%, figure 2, p<0·001). Gastro-intestinal/surgical abdomen was diagnosed more often in adolescents (16·2%) (10·1%, p<0·001).

Adolescents were more often classified as having bacterial disease (31·0% versus 21·6%, aOR 1·5, 95% CI 1·4-1·7) and SBI (15·8% versus 8·4%, aOR 1·8, 95%CI 1·6-2·0, and less often with probable/definite viral disease. Most common SBI in both adolescents and younger children were lower respiratory tract, urinary tract and gastro-intestinal infections. Bacterial sepsis/meningitis was more common in adolescents (0·6% versus 0·3%, OR 1·9 (1·1-3·3)), although after adjusting for general patient characteristics, this was significant only in children without comorbidity (aOR 2·3, 95% CI 1·1-5·0, figure 3).

Presentation and management of children and adolescents with SBI.

In total 3,347 children presented with SBI. SBI was present in 406 of 2,577 adolescents (15,8%) and 2,941 of 34,843 of younger children (8,4%).

Adolescents with SBI more often had comorbidity (34·0% versus 23·6%, p<0·001), less often presented with tachypnoea and increased work of breathing, while rates of tachycardia and

For peer review only - http://bmjopen.bmj.com/site/about/guidelines.xhtml

prolonged capillary refill were similar between adolescents and younger children with SBI. Adolescents with SBI and sepsis/meningitis were more often described as ill-appearing and adolescents that were described as ill-appearing, more often were diagnosed with SBI or sepsis/meningitis. However, high-risk criteria from the NICE sepsis guideline were present less frequently in adolescents with SBI (appendix 6).

No differences were found regarding the frequency of CRP >60mg/l, intravenous antibiotics, admission or PICU admission. Adolescents with SBI more often were treated with ILSI than younger children (aOR 2·2, 95% CI 1·4-3·5), although this difference was not significant after excluding children with comorbidity.

DISCUSSION

Main findings

Our data show that despite accounting for a small fraction of all ED visits for febrile children, adolescents presenting to the ED have an increased risk of SBI such as sepsis/meningitis. Furthermore, adolescents with SBI present differently than younger children. Although adolescents were more often described as ill-appearing, high-risk criteria from the NICE sepsis guideline were present less frequently in adolescents with SBI or sepsis/meningitis. Adolescents were hospitalized more often, and more often received intravenous antibiotics and ILSI. Although adolescents more often had comorbidities, SBI was still more common in adolescents after excluding children with comorbidities.

For peer review only

Findings in relation to previous literature

Previous studies on febrile children have focused on infants and young children,[5] and literature regarding adolescents is scarce. Our data is in line with the few studies that have found an increased incidence of sepsis in adolescents in comparison to younger children[7,9] and emphasize how febrile adolescents form a distinct risk group. Previous studies[23] have suggested that there might be a delay in adolescents presenting to the ED. However, no differences were found in fever duration between adolescents and younger children diagnosed with SBI in our study.

Our data show that the *health paradox* does not only apply to preventable injuries, but also to potentially preventable infectious diseases.

Implications for clinical practice and research

Health care workers evaluating adolescents should be aware of the substantial risk in adolescents for SBI and the potentially different presentation. Diagnostic tests and antibiotic therapy should be considered when SBI cannot be ruled out on clinical grounds and safety netting advice should be given to all febrile adolescents and their caregivers in case of discharge from the ED. Currently, there is a lack of guidelines on the management of febrile adolescents, as, although the NICE sepsis guideline addresses adolescents as well as younger children,[15] its focus is on the recognition of sepsis, while the more general NICE fever guideline is exclusively targeted at children below the age of five.[15] Based on our data, it would be advisable to develop a more general guideline that aids in the management of febrile adolescents and includes (clinical) markers for the recognition of SBI other than sepsis.

For peer review only - http://bmjopen.bmj.com/site/about/guidelines.xhtml

As adolescents with SBI less often presented with the classical high-risk criteria cited in the NICE sepsis guideline, future research should focus on identifying alarming signs in the adolescent age-group.

Secondly, empowering caregivers is an important step in the care for their febrile child,[24] as most studies regarding caregivers' knowledge on fever have focused on young children,[25] as do many online information sources for caregivers.[26]

STRENGTHS AND LIMITATIONS

To our knowledge this is the first study looking into patient characteristics, management and diagnosis of febrile adolescents attending the ED.

The main strengths of our study are that detailed information on presenting signs, management and diagnosis was collected on a large number of children and adolescents in different European EDs. Data were collected year-round and included different hospitals with different patient case mixes, which largely increases the generalizability of the results.[14,27]

Furthermore, we included a large number of children with SBI, as determined by a uniformly applied flowchart.

The main limitations include the lack of information regarding outcome after the ED visit, e.g. revisits, 30-day morbidity and mortality and the use of routinely collected data. To ensure data quality, all study sites were extensively trained regarding the accurate documentation of patient characteristics and quality checks were performed regularly. The amount of missing data was limited and its effects were reduced by using multiple imputation.[28]

For peer review only - http://bmjopen.bmj.com/site/about/guidelines.xhtml

Another limitation is that blood pressure, cyanosis and diminished urine output were not included in the data collection. A previous study showed that although hypotension is associated with serious illness in children, its sensitivity is limited as routine measurement in *all* children attending the ED.[29,30] and it is a late sign in septic children in comparison to adults. On the other hand, in adolescents, similar to adults, hypotension might present earlier in the disease course, and thus including blood pressure could provide valuable information.

As cases defined as 'probable bacterial' were also included in the definition of sepsis/meningitis, we cannot preclude that some of these cases were not of bacterial origin in either age-groups. However, in the EUCLIDS study on severe sepsis, a pathogen was only found in half of the cases.[11]

Lastly, our data applies to adolescents attending the ED; more research is needed to know whether our results can be applied to adolescents presenting to primary care as well. Furthermore, it is unknown whether a form of "selection bias" exists, as parents might be more inclined to seek help for younger febrile children than for adolescents.

CONCLUSION

Our data show that despite accounting for a relatively small fraction of all ED visits, febrile adolescents have an increased risk of serious bacterial infections including sepsis/meningitis, in comparison to younger children.

For peer review only - http://bmjopen.bmj.com/site/about/guidelines.xhtml

References

1. Willoughby T, Good M, Adachi PJ, et al. Examining the link between adolescent brain development and risk taking from a social-developmental perspective (reprinted). *Brain Cogn* 2014,Aug;89:70-8.
2. Bell TM, Qiao N, Jenkins PC, et al. Trends in emergency department visits for nonfatal violence-related injuries among adolescents in the united states, 2009-2013. *J Adolesc Health* 2016;58(5):573-5.
3. Lieberman A, Badolato GM, Tran J, et al. Frequency of prescription filling among adolescents prescribed treatment for sexually transmitted infections in the emergency department. *JAMA Pediatr* 2019,Jul 1;173(7):695-7.
4. Lee J, Bang YS, Min S, et al. Characteristics of adolescents who visit the emergency department following suicide attempts: Comparison study between adolescents and adults. *BMC Psychiatry* 2019,Jul 26;19(1):231.
5. Cioffredi LA, Jhaveri R. Evaluation and management of febrile children: A review. *JAMA Pediatr* 2016;170(8):794-800.
6. Angus DC, Linde-Zwirble WT, Lidicker J, et al. Epidemiology of severe sepsis in the united states: Analysis of incidence, outcome, and associated costs of care. *Crit Care Med* 2001,Jul;29(7):1303-10.
7. Watson RS, Carcillo JA, Linde-Zwirble WT, et al. The epidemiology of severe sepsis in children in the united states. *Am J Respir Crit Care Med* 2003,Mar 1;167(5):695-701.
8. Davis KL, Bell TJ, Miller JM, et al. Hospital costs, length of stay and mortality associated with childhood, adolescent and young adult meningococcal disease in the US. *Appl Health Econ Health Policy* 2011,May 1;9(3):197-207.

For peer review only - http://bmjopen.bmj.com/site/about/guidelines.xhtml

9. Campbell H, Parikh SR, Borrow R, et al. Presentation with gastrointestinal symptoms and high case fatality associated with group W meningococcal disease (menw) in teenagers, england, july 2015 to january 2016. *Euro Surveill* 2016;21(12).

10. Burman C, Serra L, Nuttens C, et al. Meningococcal disease in adolescents and young adults: A review of the rationale for prevention through vaccination. *Hum Vaccin Immunother* 2019;15(2):459-69.

11. Boeddha NP, Schlapbach LJ, Driessen GJ, et al. Mortality and morbidity in community-acquired sepsis in european pediatric intensive care units: A prospective cohort study from the european childhood life-threatening infectious disease study (EUCLIDS). *Crit Care* 2018;22(1):143.

12. Thompson MJ, Ninis N, Perera R, et al. Clinical recognition of meningococcal disease in children and adolescents. *Lancet* 2006,Feb 4;367(9508):397-403.

13. https://www.perform2020.org/.

14. Hagedoorn NN, Borensztajn DM, Nijman R, et al. Variation in antibiotic prescription rates in febrile children presenting to emergency departments across europe (MOFICHE): A multicentre observational study. *PLoS Med* 2020;17(8):e1003208.

15. https://www.nice.org.uk.

16. Simon TD, Cawthon ML, Stanford S, et al. Pediatric medical complexity algorithm: A new method to stratify children by medical complexity. *Pediatrics* 2014,Jun;133(6):e1647-54.

17. Turner NM. Advanced paediatric life support: Dutch Edition; 2017.

18. Parshuram CS, Hutchison J, Middaugh K. Development and initial validation of the bedside paediatric early warning system score. *Crit Care* 2009;13(4):R135.

19. Vredebregt SJ, Moll HA, Smit FJ, et al. Recognizing critically ill children with a modified pediatric early warning score at the emergency department, a feasibility study. *Eur J Pediatr* 2019,Feb;178(2):229-34.

20. Lee JY, Oh SH, Peck EH, et al. The validity of the canadian triage and acuity scale in predicting resource utilization and the need for immediate life-saving interventions in

For peer review only

For peer review only - http://bmjopen.bmj.com/site/about/guidelines.xhtml

elderly emergency department patients. *Scand J Trauma Resusc Emerg Med* 2011,Nov 3;19:68.

21. Herberg JA, Kaforou M, Wright VJ, et al. Diagnostic test accuracy of a 2-transcript host RNA signature for discriminating bacterial vs viral infection in febrile children. *JAMA* 2016;316(8):835-45.
22. Martinón-Torres F, Salas A, Rivero-Calle I, et al. Life-threatening infections in children in europe (the EUCLIDS project): A prospective cohort study. L*ancet Child Adolesc Health* 2018;2(6):404-14.
23. Ziv A, Boulet JR, Slap GB. Emergency department utilization by adolescents in the united states. *Pediatrics* 1998,Jun;101(6):987-94.
24. van de Maat JS, van Klink D, den Hartogh-Griffioen A, et al. Development and evaluation of a hospital discharge information package to empower parents in caring for a child with a fever. *BMJ Open* 2018;8(8):e021697.
25. Thompson AP, Nesari M, Hartling L, et al. Parents' experiences and information needs related to childhood fever: A systematic review. *Patient Educ Couns* 2020,Apr;103(4):750-63.
26. https://what0-18.nhs.uk/professionals/pharmacists/safety-netting-documents-parents/fever-children-under-5-years.
27. Borensztajn D, Yeung S, Hagedoorn NN, et al. Diversity in the emergency care for febrile children in europe: A questionnaire study. *BMJ Paediatr Open* 2019;3(1):e000456.
28. Vergouwe Y, Royston P, Moons KG, et al. Development and validation of a prediction model with missing predictor data: A practical approach. *J Clin Epidemiol* 2010,Feb;63(2):205-14.
29. Hagedoorn NN, Zachariasse JM, Moll HA. Association between hypotension and serious illness in the emergency department: An observational study. *Arch Dis Child* 2020,Jun;105(6):545-551.

For peer review only

30. Stewart JN, McGillivray D, Sussman J, et al. The value of routine blood pressure measurement in children presenting to the emergency department with nonurgent problems. *J Pediatr* 2008,Oct;153(4):478-83.

Tables and figures

For peer review only

Table 1: Differences in patient characteristics between young children and adolescents (n=37,420)

	Children 3 months- 12 years N = 34,843 N (%)	Children ≥ 12 years N = 2,577 N (%)
**Male**	19,182 (55·1)	1,307 (50·7)
**Age in years, median (IQR)**	2·6 (1·3-4·9)	14·5 (13·2-16·1)
**Comorbidity**[a]		
**Simple**	4,302 (12·5)	489 (19·1)
**Complex**	1,332 (3·9)	241 (9·4)
**Duration of fever** *		
**< 24 hours**	11,410 (35·1)	854 (37,3)
**24-48 hours**	10,622 (32,7)	682 (29,8)
**> 48 hours**	10,433 (31,1)	755 (33,0)
**Referral**		
**Self**	19,537 (57·8)	1,231 (49·4)
**GP/private paediatrician**	5,654 (16·7)	493 (19·8)
**Emergency medical service**	5,010 (14·8)	430 (17·3)
**Other**	3,574 (10·6)	337 (13·5)
**Triage urgency**		
**High: immediate, very urgent, intermediate**	11,664 (34·5)	967 (38·9)
**Vital signs**[b] **and PEWS**		
**Tachycardia APLS**	8,552 (24·5)	764 (29·6)
**Tachypnoea APLS**	5,282 (15·2)	189 (7)
**Hypoxia, oxygen saturation <95% APLS**	805 (2·3)	30 (1)
**Prolonged capillary refill >=3 seconds**[ns]	343 (1·1)	25 (1)
**Simplified PEWS 6 or higher**	782 (4·5)	81 (6)
**Nice "red traffic lights" (alarming signs)**		
**Ill appearance**	5,203 (15·6)	559 (23·1)
**Increased work of breathing**	3,050 (10·0)	67 (3)
**Rash: petechiae/non-blanching**	1,040 (3·4)	53 (2)
**Decreased consciousness**[ns]	178 (1)	16 (1)
**Meningeal signs**	97 (0)	23 (1)
**Status epilepticus**[ns]	58 (0)	8 (0)
**Focal neurology**	110 (0)	19 (1)

Missing values: general patient characteristics: < 7%. Vital signs: 9-23%. NICE alarming signs 1-18%.
[a]. Comorbidity: a chronic underlying condition that is expected to last at least 1 year. Complex comorbidity: a chronic condition in ≥2 body systems or malignancy or immunocompromised patients.
[b]. According to APLS cut-off values by age.
All comparisons p<0·001, except * = <0·05 and [ns] = not significant.

For peer review only - http://bmjopen.bmj.com/site/about/guidelines.xhtml

Table 2. "Red traffic light" symptoms (alarming signs) from the NICE guideline on fever and high-risk criteria from the NICE sepsis guideline.

	Fever < 5[a]	Sepsis < 5[b]	Sepsis 5-11[b]	Sepsis ≥12[b]
**Behaviour**				
No response to social cues[c]	+	+		
Altered behaviour[c]			+	+
Ill appearance	+	+	+	
Does not wake / does not stay awake	+	+	+	
Weak high-pitched or continuous cry[d]	+	+		
**Respiratory**				
Grunting	+	+		
Apnoea		+		
Oxygen saturation < 90%		+	+	
Oxygen saturation < 93%				+
Tachypnoea for age	+	+	+	+
Chest retractions	+			
**Circulation**				
Bradycardia < 60		+	+	
Tachycardia for age		+	+	+
Reduced skin turgor	+			
Not passed urine in previous 18 hours.				+
Systolic blood pressure 90 mmHg				+
**Skin**				
Mottled, ashen or cyanosis	+	+	+	+
Non-blanching rash	+	+	+	+
**Temperature**				
< 36·0° C		+		
≥38·0 in infants < 3 months	+	+		
**Neurological**				
Bulging fontanelle or neck stiffness	+			
Status epilepticus	+			
Focal neurological signs	+			
Focal seizures	+			
		Data available	Available (proxy used)	Not available

[a] NICE fever guideline
[b] NICE sepsis guideline
[c] Defined as reduced consciousness
[d] defined as ill appearance

For peer review only - http://bmjopen.bmj.com/site/about/guidelines.xhtml

Figure 1: Adjusted odds ratios for younger children versus adolescents for diagnostic tests, therapy, disposition and final diagnosis. [a]

[a] Younger children used as reference. Adjusted for hospital, sex, duration of fever, previous medical care, time of arrival and comorbidity.

To convert CRP values to nmol/L, multiply by 0·9524

For peer review only

Figure 2: focus of infection in young children and adolescents

Data shown as percentages within the groups of young children and adolescents.
LRTI=lower respiratory tract infection; gastro-intestinal=gastro-intestinal and surgical abdomen; UTI=urinary tract infection, exanthems=exanthems and flulike illness; musculoskeletal=soft-tissue, skin and musculoskeletal infection.
LRTI (not shown in graphic) = lower respiratory tract infection: young children 54%, adolescents 42%.

For peer review only

Figure 3: Adjusted odds ratios for younger children versus adolescents for diagnostic tests, therapy, disposition and final diagnosis, patients with comorbidity excluded.[a]

[a] Younger children used as reference. Adjusted for hospital, sex, duration of fever, previous medical care and time of arrival.

To convert CRP values to nmol/L, multiply by 0·9524

For peer review only

For peer review only - http://bmjopen.bmj.com/site/about/guidelines.xhtml

Acknowledgments

We gratefully acknowledge the emergency department staff of the participating hospitals for their participation and collection of data. In addition, we thank Arianne van Rijn, (former) medical student for her assistance with the literature review.

For peer review only

For peer review only - http://bmjopen.bmj.com/site/about/guidelines.xhtml

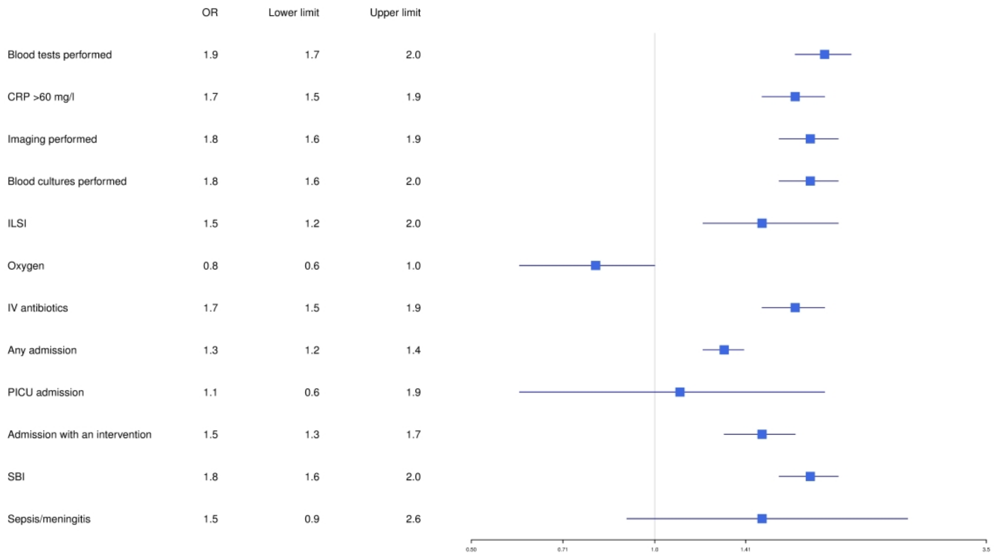

396x233mm (600 x 600 DPI)

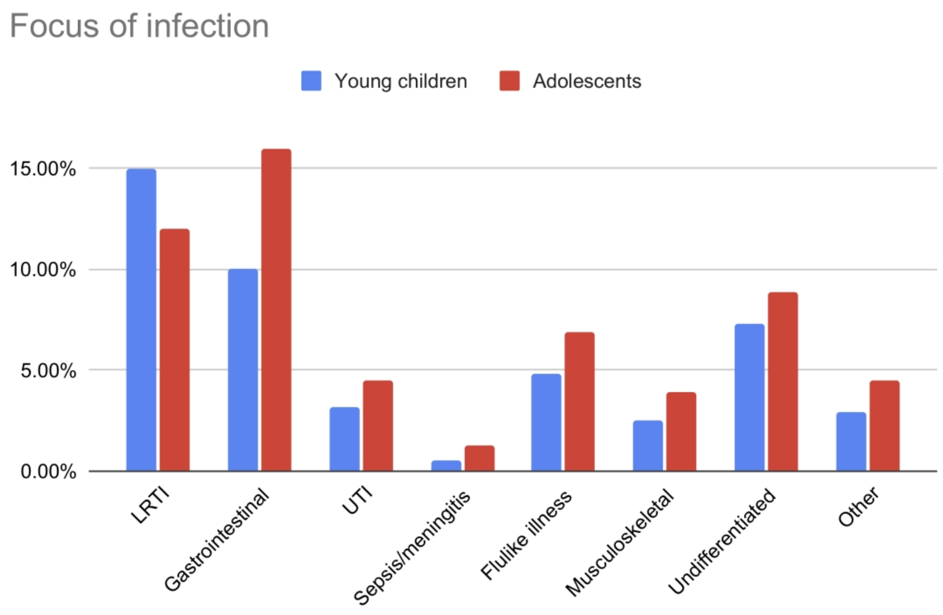

211x153mm (300 x 300 DPI)

	OR	Lower limit	Upper limit
Blood tests performed	1.7	1.5	1.9
CRP > 60 mg/l	1.5	1.3	1.8
Imaging performed	1.6	1.5	1.8
Blood cultures performed	1.5	1.3	1.8
ILSI	1.2	0.7	1.9
Oxygen	0.3	0.2	0.6
IV antibiotics	1.7	1.5	2.0
Any admission	1.3	1.1	1.4
PICU admission	1.0	0.3	2.6
Admission with an intervention	1.4	1.2	1.7
SBI	1.8	1.6	2.1
Sepsis/meningitis	2.3	1.1	5.0

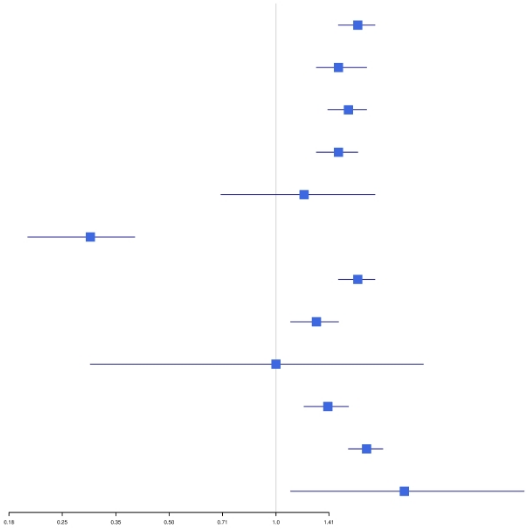

396x233mm (600 x 600 DPI)

PERFORM consortium author list.

PARTNER: IMPERIAL COLLEGE (UK)

Chief investigator/PERFORM coordinator:

Michael Levin

Principal and co-investigators; work package leads (alphabetical order)

Aubrey Cunnington (grant application)

Tisham De (work package lead)

Jethro Herberg (Principle Investigator, Deputy Coordinator, grant application)

Myrsini Kaforou (grant application, work package lead)

Victoria Wright (grant application, Scientific Coordinator)

Research Group (alphabetical order)

Lucas Baumard; Evangelos Bellos; Giselle D'Souza; Rachel Galassini; Dominic Habgood-Coote; Shea Hamilton; Clive Hoggart; Sara Hourmat; Heather Jackson; Ian Maconochie; Stephanie Menikou; Naomi Lin; Samuel Nichols; Ruud Nijman; Ivonne Pena Paz; Priyen Shah; Ching-Fen Shen; Ortensia Vito; Clare Wilson

Clinical recruitment at Imperial College Healthcare NHS Trust (alphabetical order))

Amina Abdulla; Ladan Ali; Sarah Darnell; Rikke Jorgensen; Sobia Mustafa; Salina Persand

Imperial College Faculty of Engineering

Molly Stevens (co-investigator), Eunjung Kim (research group); Benjamin Pierce (research group)

Clinical recruitment at Brighton and Sussex University Hospitals

Katy Fidler (Principle Investigator)

Julia Dudley (Clinical Research Registrar)

Research nurses: Vivien Richmond, Emma Tavliavini

Clinical recruitment at National Cheng Kung University Hospital

Ching-Fen Shen (Principal Investigator); Ching-Chuan Liu (Co-investigator); Shih-Min Wang (Co-investigator), funded by the Center of Clinical Medicine Research, National Cheng Kung University

For peer review only - http://bmjopen.bmj.com/site/about/guidelines.xhtml

SERGAS Partner (Spain)

Principal Investigators

Federico Martinón-Torres[1]

Antonio Salas[1,2]

GENVIP RESEARCH GROUP (in alphabetical order):

Fernando Álvez González[1], Cristina Balo Farto[1], Ruth Barral-Arca[1,2], María Barreiro Castro[1], Xabier Bello[1,2], Mirian Ben García[1], Sandra Carnota[1], Miriam Cebey-López[1], María José Curras-Tuala[1,2], Carlos Durán Suárez[1], Luisa García Vicente[1], Alberto Gómez-Carballa[1,2], Jose Gómez Rial[1], Pilar Leboráns Iglesias[1], Federico Martinón-Torres[1], Nazareth Martinón-Torres[1], José María Martinón Sánchez[1], Belén Mosquera Pérez[1], Jacobo Pardo-Seco[1,2], Lidia Piñeiro Rodríguez[1], Sara Pischedda[1,2], Sara Rey Vázquez[1], Irene Rivero Calle[1], Carmen Rodríguez-Tenreiro[1], Lorenzo Redondo-Collazo[1], Miguel Sadiki Ora[1], Antonio Salas[1,2], Sonia Serén Fernández[1], Cristina Serén Trasorras[1], Marisol Vilas Iglesias[1].

[1] Translational Pediatrics and Infectious Diseases, Pediatrics Department, Hospital Clínico Universitario de Santiago, Santiago de Compostela, Spain, and GENVIP Research Group (www.genvip.org), Instituto de Investigación Sanitaria de Santiago, Universidad de Santiago de Compostela, Galicia, Spain.

[2] Unidade de Xenética, Departamento de Anatomía Patolóxica e Ciencias Forenses, Instituto de Ciencias Forenses, Facultade de Medicina, Universidade de Santiago de Compostela, and GenPop Research Group, Instituto de Investigaciones Sanitarias (IDIS), Hospital Clínico Universitario de Santiago, Galicia, Spain

[3] Fundación Pública Galega de Medicina Xenómica, Servizo Galego de Saúde (SERGAS), Instituto de Investigaciones Sanitarias (IDIS), and Grupo de Medicina Xenómica, Centro de Investigación Biomédica en Red de Enfermedades Raras (CIBERER), Universidade de Santiago de Compostela (USC), Santiago de Compostela, Spain

For peer review only

For peer review only - http://bmjopen.bmj.com/site/about/guidelines.xhtml

RSU Partner (Latvia)

Principal Investigator

Dace Zavadska[1,2]

Other RSU group authors (in alphabetical order):

Anda Balode[1,2], Arta Bārzdiņa[1,2], Dārta Deksne[1,2], Dace Gardovska[1,2], Dagne Grāvele[2], Ilze Grope[1,2], Anija Meiere[1,2], Ieva Nokalna[1,2], Jana Pavāre[1,2], Zanda Pučuka[1,2], Katrīna Selecka[1,2], Aleksandra Sidorova[1,2], Dace Svile[2], Urzula Nora Urbāne[1,2].

[1] Riga Stradins university, Riga, Latvia.

[2] Children clinical university hospital, Riga, Latvia.

For peer review only

Medical Research Council Unit The Gambia (MRCG) at LSHTM Partner

Principal Investigator
Effua Usuf

Additional Investigators
Kalifa Bojang
Syed M. A. Zaman
Fatou Secka
Suzanne Anderson
Anna Rocalsatou Sarr
Momodou Saidykhan
Saffiatou Darboe
Samba Ceesay
Umberto D’alessandro

Medical Research Council Unit The Gambia at LSHTM
P O Box 273,
Fajara, The Gambia

For peer review only

ERASMUS MC-Sophia Children's Hospital

Principal Investigator

Henriëtte A. Moll[1]

Research group

Dorine M. Borensztajn[1], Nienke N. Hagedoorn, Chantal Tan [1], [1], Clementien L. Vermont[2], Joany Zachariasse [1]

Additional investigator

W Dik [3]

[1] Erasmus MC-Sophia Children's Hospital, Department of General Paediatrics, Rotterdam, the Netherlands

[2] Erasmus MC-Sophia Children's Hospital, Department of Paediatric Infectious Diseases & Immunology, Rotterdam, the Netherlands

[3] Erasmus MC, Department of immunology, Rotterdam, the Netherlands

For peer review only

Swiss Pediatric Sepsis Study

Principal Investigators*:*

Philipp Agyeman, MD [1] (ORCID 0000-0002-8339-5444), Luregn J Schlapbach, MD, FCICM [2,3] (ORCID 0000-0003-2281-2598)

Clinical recruitment at University Children's Hospital Bern for PERFORM:

Christoph Aebi [1], Verena Wyss [1], Mariama Usman [1]

Principal and co-investigators for the Swiss Pediatric Sepsis Study:

Philipp Agyeman, MD [1], Luregn J Schlapbach, MD, FCICM [2,3], Eric Giannoni, MD [4,5], Martin Stocker, MD [6], Klara M Posfay-Barbe, MD [7], Ulrich Heininger, MD [8], Sara Bernhard-Stirnemann, MD [9], Anita Niederer-Loher, MD [10], Christian Kahlert, MD [10], Giancarlo Natalucci, MD [11], Christa Relly, MD [12], Thomas Riedel, MD [13], Christoph Aebi, MD [1], Christoph Berger, MD [12] **for the Swiss Pediatric Sepsis Study**

Affiliations:

[1] Department of Pediatrics, Inselspital, Bern University Hospital, University of Bern, Switzerland

[2] Neonatal and Pediatric Intensive Care Unit, Children's Research Center, University Children's Hospital Zurich, University of Zurich, Zurich, Switzerland

[3]Child Health Research Centre, University of Queensland, and Queensland Children`s Hospital, Brisbane, Australia

[4] Clinic of Neonatology, Department Mother-Woman-Child, Lausanne University Hospital and University of Lausanne, Switzerland

[5] Infectious Diseases Service, Department of Medicine, Lausanne University Hospital and University of Lausanne, Switzerland

[6] Department of Pediatrics, Children's Hospital Lucerne, Lucerne, Switzerland

[7] Pediatric Infectious Diseases Unit, Children's Hospital of Geneva, University Hospitals of Geneva, Geneva, Switzerland

[8] Infectious Diseases and Vaccinology, University of Basel Children's Hospital, Basel, Switzerland

[9] Children's Hospital Aarau, Aarau, Switzerland

[10] Division of Infectious Diseases and Hospital Epidemiology, Children's Hospital of Eastern Switzerland St. Gallen, St. Gallen, Switzerland

[11] Department of Neonatology, University Hospital Zurich, Zurich, Switzerland

[12] Division of Infectious Diseases and Hospital Epidemiology, and Children's Research Center, University Children's Hospital Zurich, Switzerland

[13] Children's Hospital Chur, Chur, Switzerland

For peer review only - http://bmjopen.bmj.com/site/about/guidelines.xhtml

Liverpool Partner

Principal Investigators

Enitan D Carrol[1,2,3]

Stéphane Paulus [1,]

Research Group (in alphabetical order):

Elizabeth Cocklin[1], Rebecca Jennings[4], Joanne Johnston[4], Simon Leigh[1], Karen Newall[4], Sam Romaine[1]

[1] Department of Clinical Infection, Microbiology and Immunology, University of Liverpool Institute of Infection and Global Health , Liverpool, England

[2] Alder Hey Children's Hospital, Department of Infectious Diseases, Eaton Road, Liverpool, L12 2AP

[3] Liverpool Health Partners, 1st Floor, Liverpool Science Park, 131 Mount Pleasant, Liverpool, L3 5TF

[4]Alder Hey Children's Hospital, Clinical Research Business Unit, Eaton Road, Liverpool, L12 2AP

For peer review only - http://bmjopen.bmj.com/site/about/guidelines.xhtml

NKUA Partner (Greece)

Principal investigator: Professor **Maria Tsolia** (all activities)

Investigator/Research fellow: **Irini Eleftheriou** (all activities)

Additional investigators:

Recruitment: Maria Tambouratzi

Lab: Antonis Marmarinos (Quality Manager)

Lab: Marietta Xagorari

Kelly Syggelou

2nd Department of Pediatrics, National and Kapodistrian University of Athens,

“P. and A. Kyriakou” Children’s Hospital

Thivon and Levadias

Goudi, Athens

For peer review only

Micropathology Ltd :

Principal Investigator:

Professor Colin Fink[1], Clinical Microbiologist

Additional investigators

Dr Marie Voice[1], Post doc scientist

Dr. Leo Calvo-Bado[1], Post doc scientist

[1] Micropathology Ltd, The Venture Center, University of Warwick Science Park, Sir William Lyons Road, Coventry, CV4 7EZ.

For peer review only

Medical University of Graz, Austria (MUG)

Principal Investigator:

Werner Zenz[1] (all activities)

Co-investigators (in alphabetical order)

Benno Kohlmaier[1] (all activities)

Nina A. Schweintzger[1] (all activities)

Manfred G. Sagmeister[1] (study design, consortium wide sample management)

Research team

Daniela S. Kohlfürst[1] (study design)

Christoph Zurl[1] (BIVA PIC)

Alexander Binder[1] (grant application)

Recruitment team, data managers, (in alphabetical order):

Susanne Hösele[1], Manuel Leitner[1], Lena Pölz[1], Glorija Rajic[1],

Clinical recruitment partners (in alphabetical order):

Sebastian Bauchinger[1], Hinrich Baumgart[4], Martin Benesch[3], Astrid Ceolotto[1], Ernst Eber[2], Siegfried Gallistl[1], Gunther Gores[5], Harald Haidl[1], Almuthe Hauer[1], Christa Hude[1], Markus Keldorfer[5], Larissa Krenn[4], Heidemarie Pilch[5], Andreas Pfleger[2], Klaus Pfurtscheller[4], Gudrun Nordberg[5], Tobias Niedrist[8], Siegfried Rödl[4], Andrea Skrabl-Baumgartner[1], Matthias Sperl[7], Laura Stampfer[5], Volker Strenger[3], Holger Till[6], Andreas Trobisch[5], Sabine Löffler[5]

Author Affiliations:

[1] Department of Pediatrics and Adolescent Medicine, Division of General Pediatrics, Medical University of Graz, Graz, Austria

[2]Department of Pediatric Pulmonology, Medical University of Graz, Graz, Austria

[3]Department of Pediatric Hematooncoloy, Medical University of Graz, Graz, Austria

[4]Paediatric Intensive Care Unit, Medical University of Graz, Graz, Austria

For peer review only - http://bmjopen.bmj.com/site/about/guidelines.xhtml

[5]University Clinic of Paediatrics and Adolescent Medicine Graz, Medical University Graz, Graz,Austria

[6]Department of Paediatric and Adolescence Surgery, Medical University Graz, Graz, Austria

[7]Department of Pediatric Orthopedics, Medical University Graz, Graz, Austria

[8]Clinical Institute of Medical and Chemical Laboratory Diagnostics, Medical University Graz, Graz, Austria

For peer review only

For peer review only - http://bmjopen.bmj.com/site/about/guidelines.xhtml

London School of Hygiene and Tropical Medicine

WP 1 WP2, WP5

Principal Investigator:

Dr Shunmay Yeung[1,2 3] PhD, MBBS, FRCPCH, MRCP, DTM&H

Research Group

Dr Juan Emmanuel Dewez[1] MD, DTM&H, MSc

Prof Martin Hibberd [1] BSc, PhD

Mr David Bath[2] MSc, MAppFin, BA(Hons)

Dr Alec Miners[2] BA(Hons), MSc, PhD

Dr Ruud Nijman[3] PhD MSc MD MRCPCH

Dr Catherine Wedderburn[1] BA, MBChB, DTM&H, MSc, MRCPCH

Ms Anne Meierford[1] MSc, BMedSc, BMBS

Dr Baptiste Leurent[4], PhD, MSc

1. Faculty of Infectious and Tropical Disease, London School of Hygiene and Tropical Medicine, London, UK
2. Faculty of Public Health and Policy, London School of Hygiene and Tropical Medicine, London, UK
3. Department of Paediatrics, St. Mary's Hospital Imperial College Hospital, London, UK
4. Faculty of Epidemiology and Population Health, London School of Hygiene and Tropical Medicine, London, UK

For peer review only - http://bmjopen.bmj.com/site/about/guidelines.xhtml

Radboud University Medical Center (RUMC), The Netherlands

Principal Investigators:
Ronald de Groot [1], Michiel van der Flier [1,2,3], Marien I. de Jonge[1]

Co-investigators Radboud University Medical Center (in alphabetical order):
Koen van Aerde[1,2], Wynand Alkema[1], Bryan van den Broek[1], Jolein Gloerich[1], Alain J. van Gool[1], Stefanie Henriet[1,2], Martijn Huijnen[1], Ria Philipsen[1], Esther Willems[1]

Investigators PeDBIG PERFORM DUTCH CLINICAL NETWORK (in alphabetical order):
G.P.J.M. Gerrits[8], M. van Leur[8,] J. Heidema [4],L. de Haan[1,2] C.J. Miedema [5], C. Neeleman [1] C.C. Obihara [6], G.A. Tramper-Stranders7[6]

1. Radboud University Medical Center, Nijmegen, The Netherlands
2. Amalia Children's Hospital, Nijmegen, The Netherlands
3. Wilhelmina Children's Hospital, University Medical Center Utrecht, Utrecht, The Netherlands
4. St. Antonius Hospital, Nieuwegein, The Netherlands
5. Catharina Hospital, Eindhoven, The Netherlands
6. ETZ Elisabeth, Tilburg, The Netherlands
7. Franciscus Gasthuis, Rotterdam, The Netherlands
8. Canisius Wilhelmina Hospital, Nijmegen, The Netherlands

For peer review only

For peer review only - http://bmjopen.bmj.com/site/about/guidelines.xhtml

Oxford team (UK)

Principal Investigators

Andrew J. Pollard[1,2], Rama Kandasamy[1,2], Stéphane Paulus [1,2]

Additional Investigators

Michael J. Carter[1,2], Daniel O'Connor[1,2], Sagida Bibi[1,2], Dominic F. Kelly[1,2], Meeru Gurung[3], Stephen Thorson[3], Imran Ansari[3], David R. Murdoch[4], Shrijana Shrestha[3], Zoe Oliver[5]

Author Affiliations:

[1]Oxford Vaccine Group, Department of Paediatrics, University of Oxford, Oxford, United Kingdom.

[2]NIHR Oxford Biomedical Research Centre, Oxford, United Kingdom.

[3]Paediatric Research Unit, Patan Academy of Health Sciences, Kathmandu, Nepal.

[4]Department of Pathology, University of Otago, Christchurch, New Zealand.

[5] Department of Paediatrics, University of Oxford.

For peer review only

Newcastle University, Newcastle upon Tyne, (UK)

Principal Investigator:

Marieke Emonts [1,2,3] (all activities)

Co-investigators

Emma Lim[2,3,7] (all activities)

Lucille Valentine[4]

Recruitment team (alphabetical), data-managers, and GNCH Research unit:

Karen Allen[5], Kathryn Bell[5], Adora Chan[5], Stephen Crulley[5], Kirsty Devine[5], Daniel Fabian[5], Sharon King[5], Paul McAlinden[5], Sam McDonald[5], Anne McDonnell2,[5], Ailsa Pickering[2,5], Evelyn Thomson[5], Amanda Wood[5], Diane Wallia[5], Phil Woodsford[5],

Sample processing: Frances Baxter[5], Ashley Bell[5], Mathew Rhodes[5]

PICU recruitment

Rachel Agbeko[8]

Christine Mackerness[8]

Students MOFICHE

Bryan Baas[2], Lieke Kloosterhuis[2], Wilma Oosthoek[2]

Students/medical staff PERFORM

Tasnim Arif[6], Joshua Bennet[2], Kalvin Collings[2], Ilona van der Giessen[2], Alex Martin[2], Aqeela Rashid[6], Emily Rowlands[2], Gabriella de Vries[2], Fabian van der Velden[2]

Engagement work/ethics/cost effectiveness

Lucille Valentine [4], Mike Martin[9], Ravi Mistry[2], Lucille Valentine[4]

Author Affiliations:

[1] Translational and Clinical Research Institute, Newcastle University, Newcastle upon Tyne UK

[2]Great North Children's Hospital, Paediatric Immunology, Infectious Diseases & Allergy, Newcastle upon Tyne Hospitals NHS Foundation Trust, Newcastle upon Tyne, United Kingdom.

For peer review only - http://bmjopen.bmj.com/site/about/guidelines.xhtml

[3]NIHR Newcastle Biomedical Research Centre based at Newcastle upon Tyne Hospitals NHS Trust and Newcastle University, Westgate Rd, Newcastle upon Tyne NE4 5PL, United Kingdom

[4]Newcastle University Business School, Centre for Knowledge, Innovation, Technology and Enterprise (KITE), Newcastle upon Tyne, United Kingdom

[5]Great North Children's Hospital, Research Unit, Newcastle upon Tyne Hospitals NHS Foundation Trust, Newcastle upon Tyne, United Kingdom.

[6]Great North Children's Hospital, Paediatric Oncology, Newcastle upon Tyne Hospitals NHS Foundation Trust, Newcastle upon Tyne, United Kingdom.

[7]Population Health Sciences Institute, Newcastle University, Newcastle upon Tyne, UK

[8]Great North Children's Hospital, Paediatric Intensive Care Unit, Newcastle upon Tyne Hospitals NHS Foundation Trust, Newcastle upon Tyne, United Kingdom.

[9]Northumbria University, Newcastle upon Tyne, United Kingdom.

For peer review only

For peer review only - http://bmjopen.bmj.com/site/about/guidelines.xhtml

LMU Munich Partner (Germany)

Principal Investigator:

Ulrich von Both[1,2] MD, FRCPCH (all activities)

Research group:

Laura Kolberg[1] MSc (all activities)

Manuela Zwerenz[1] MSc, Judith Buschbeck[1] PhD

Clinical recruitment partners (in alphabetical order):

Christoph Bidlingmaier[3], Vera Binder[4], Katharina Danhauser[5], Nikolaus Haas[10], Matthias Griese[6], Tobias Feuchtinger[4], Julia Keil[9], Matthias Kappler[6], Eberhard Lurz[7], Georg Muench[8], Karl Reiter[9], Carola Schoen[9]

Author Affiliations:

[1]Div. Paediatric Infectious Diseases, Hauner Children's Hospital, University Hospital, Ludwig Maximilians University (LMU), Munich, Germany

[2]German Center for Infection Research (DZIF), Partner Site Munich, Munich, Germany

[3]Div. of General Paediatrics, [4]Div. Paediatric Haematology & Oncology, [5]Div. of Paediatric Rheumatology, [6]Div. of Paediatric Pulmonology, [7]Div. of Paediatric Gastroenterology, [8]Neonatal Intensive Care Unit, [9]Paediatric Intensive Care Unit Hauner Children's Hospital, University Hospital, Ludwig Maximilians University (LMU), Munich, Germany, [10]Department Pediatric Cardiology and Pediatric Intensive Care, University Hospital, Ludwig Maximilians University (LMU), Munich, Germany

For peer review only - http://bmjopen.bmj.com/site/about/guidelines.xhtml

bioMérieux, France

Principal Investigator:
François Mallet[1,2, 3]

Research Group:
Karen Brengel-Pesce[1,2, 3]
Alexandre Pachot[1]
Marine Mommert[1,2]

[1]Open Innovation & Partnerships (OIP), bioMérieux S.A., Marcy l'Etoile, France
[2] Joint research unit Hospice Civils de Lyon - bioMérieux, Centre Hospitalier Lyon Sud, 165 Chemin du Grand Revoyet, 69310 Pierre-Bénite, France
[3] EA 7426 Pathophysiology of Injury-induced Immunosuppression, University of Lyon1-Hospices Civils de Lyon-bioMérieux, Hôpital Edouard Herriot, 5 Place d'Arsonval, 69437 Lyon Cedex 3, France

Department of Infectious Diseases, University Medical Centre Ljubljana, Slovenia

Principal Investigator:

Marko Pokorn[1,2,3] MD, PhD

Research Group:

Mojca Kolnik[1] MD, Katarina Vincek[1] MD, Tina Plankar Srovin[1] MD, PhD, Natalija Bahovec[1] MD, Petra Prunk[1] MD, Veronika Osterman[1] MD, Tanja Avramoska[1] MD

Affiliations:

[1]Department of Infectious Diseases, University Medical Centre Ljubljana, Japljeva 2, SI-1525 Ljubljana, Slovenia

[2]University Childrens' Hospital, University Medical Centre Ljubljana, Ljubljana, Slovenia

[3]Department of Infectious Diseases and Epidemiology, Faculty of Medicine, University of Ljubljana, Slovenia

For peer review only - http://bmjopen.bmj.com/site/about/guidelines.xhtml

Amsterdam, Academic Medical Hospital & Sanquin Research Institute (NL)

Principal Investigator:

Taco Kuijpers [1,2]

Co-investigators

Ilse Jongerius [2]

Recruitment team (EUCLIDS, PERFORM):

J.M. van den Berg[1], D. Schonenberg[1], A.M. Barendregt[1], D. Pajkrt[1], M. van der Kuip[1,3], A.M. van Furth[1,3]

Students PERFORM

Evelien Sprenkeler [2], Judith Zandstra [2],

Technical support PERFORM

G. van Mierlo [2], J. Geissler [2]

Author Affiliations:

[1] Amsterdam University Medical Center (Amsterdam UMC), location Academic Medical Center (AMC), Dept of Pediatric Immunology, Rheumatology and Infectious Diseases, University of Amsterdam, Amsterdam, the Netherlands

[2] Sanquin Research Institute, & Landsteiner Laboratory at the AMC, University of Amsterdam, Amsterdam, the Netherlands.

[3] Amsterdam University Medical Center (Amsterdam UMC), location Vrije Universiteit Medical Center (VUMC), Dept of Pediatric Infectious Diseases and Immunology, Free University (VU), Amsterdam, the Netherlands (former affiliation)

For peer review only

For peer review only - http://bmjopen.bmj.com/site/about/guidelines.xhtml

Appendix 2: Participating EDs

Hospital	Country, city	Hospital type	Total annual paediatric ED visits	Total period of inclusion	Period of inclusion per month	Number of patients included
Medizinische Universität Graz	Austria, Graz	University	10,000-30,000	1-1-2017 – 31-12-2018	10 days	2243
Dr. von Hauner Children's Hospital	Germany, Munich	Teaching	10,000-30,000	1-1-2017 – 31-12-2018	1 week	1175
P. and A. Kyriakou Children's Hospital	Greece, Athens	University	>30,000	1-1-2017 – 1-5-2018	1-2 weeks	4549
Children clinical university hospital	Latvia, Riga	Teaching	>30,000	1-1-2017 – 31-12-2018	All	9000
Univerzitetni Klinični Center	Slovenia, Ljubljana	University	<10,000	1-1-2017 – 31-12-2018	All	3659
Hospital Clínico Universitario	Spain, Santiago de Compostela	University	>30,000	1-1-2017 – 1-5-2018	1-2 weeks	3877
Erasmus MC-Sophia Children's Hospital	The Netherlands, Rotterdam	University	<10,000	1-1-2017 – 1-4-2018	All	1681
RadboudUMC	The Netherlands, Nijmegen	University	<10,000	1-1-2017 – 1-4-2018	All	676
Canisius Wilhelmina Ziekenhuis	The Netherlands, Nijmegen	Teaching	<10,000	1-1-2017 – 31-12-2018	2 weeks	415
Alder Hey Children's Hospital	United Kingdom, Liverpool	Teaching	>30,000	1-1-2017 – 31-12-2018	1 week	1624
St. Mary's Hospital	United Kingdom, London	University	10,000-30,000	1-1-2017 – 31-12-2018	All	5714
Great North Children's Hospital	United Kingdom, Newcastle upon Tyne	University	>30,000	1-4-2017 – 1-4-2018	2 weeks	3870

For peer review only

Appendix 3. Immediate life-saving interventions

1. Airway and breathing support, including intubation or emergent noninvasive positive pressure ventilation.
2. Electrical therapy, including defibrillation, emergent cardioversion, or external pacing.
3. Procedures, including chest needle decompression, pericardiocentesis, or open thoracotomy.
4. Hemodynamic support, including significant intravenous fluid resuscitation in the setting of hypotension, blood administration, or control of major bleeding.
5. Emergency medications, including naloxone, dextrose, atropine, adenosine, epinephrine, or vasopressors

For peer review only

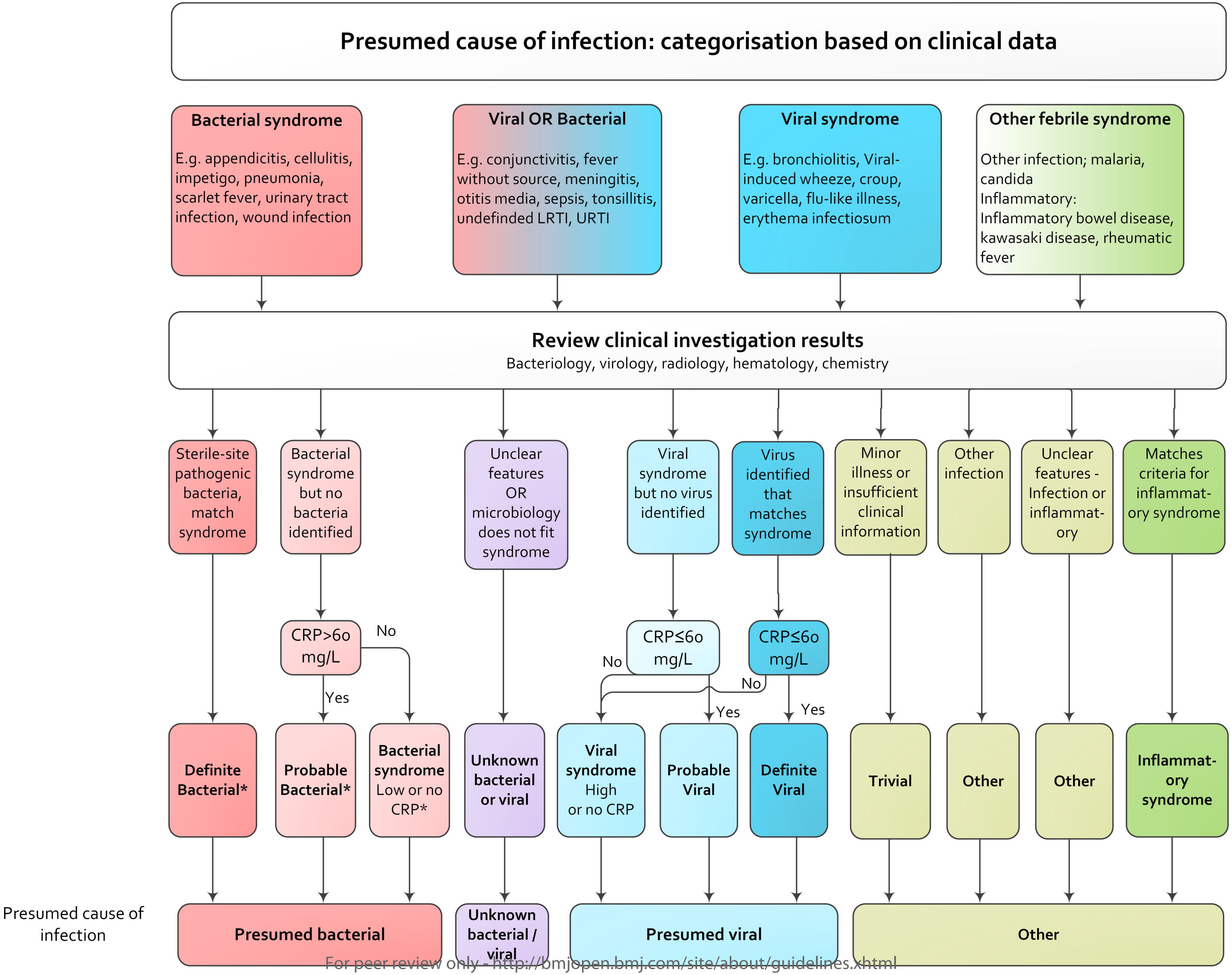

For peer review only - http://bmjopen.bmj.com/site/about/guidelines.xhtml

Appendix 5: Comorbidity.

	Children 3 months- 12 years N = 34,843 N (%)	Children > 12 years N = 2,577 N (%)
No comorbidity	28,881 (83·7)	1,833 (71·5)
Non-complex comorbidity	4,302 (12·5)	489 (19·1)
Complex comorbidity	1,332 (3·9)	241 (9·4)
Type of comorbidity		
Neurological & psychomotor delay	1,604 (28·5)	298 (40·8)
Pulmonary	1,224 (21·7)	182 (24·9)
Prematurity	945 (16·8)	20 (2·7)
Urological/nephrological	634 (11·3)	57 (7·8)
Malignancy & immunodeficiency	583 (10·4)	162 (22·2)
Cardiac	557 (9·9)	58 (7·9)
Gastro-intestinal	422 (7·5)	81 (11·1)
Musculoskeletal	171 (3·0)	55 (7·5)
Metabolic	165 (2·9)	53 (7·3)
Endocrine	63 (1·1)	27 (3·7)
Other comorbidity	729 (12·9)	83 (11·3)

Type of comorbidity displayed as percentage of children with comorbidity.

For peer review only

Appendix 6: Differences in patient characteristics between young children and adolescents with a final diagnosis of SBI. [a]

	OR	Lower limit	Upper limit
General patient characteristics			
Triage urgency high	1.0	0.8	1.3
Referral by EMS	1.0	1.0	1.1
Comorbidity	1.7	1.4	2.2
Vital signs			
Tachycardia	0.9	0.7	1.2
Tachypnoea	0.5	0.3	0.6
Prolonged capillary refill	1.1	0.6	2.2

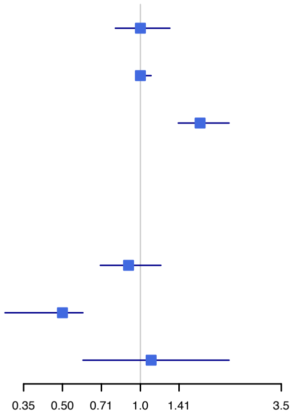

	OR	Lower limit	Upper limit
NICE fever guideline alarming signs			
Ill appearance	1.4	1.1	1.8
Non−blanching rash	0.8	0.3	2.1
Blanching rash	0.7	0.4	1.1
Increased work of breathing	0.4	0.3	0.7
Neurological signs	1.1	0.3	3.1
NICE sepsis guideline high risk criteria			
1 or more	0.4	0.3	0.5
2 or more	0.3	0.2	0.4
3 or more	0.2	0.1	0.3

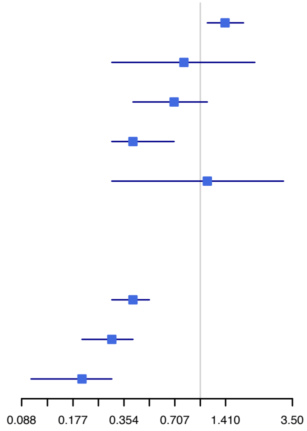

[a] Younger children used as reference. Adjusted for hospital, sex, duration of fever, previous medical care, time of arrival and comorbidity. * According to APLS cut-off values by age.

For peer review only

STROBE Statement—Checklist of items that should be included in reports of ***cohort studies***

	Item No	Recommendation	Page No
**5Title and abstract**	1	(*a*) Indicate the study's design with a commonly used term in the title or the abstract	5
		(*b*) Provide in the abstract an informative and balanced summary of what was done and what was found	5
**Introduction**			
Background/rationale	2	Explain the scientific background and rationale for the investigation being reported	7
Objectives	3	State specific objectives, including any prespecified hypotheses	7
**Methods**			
Study design	4	Present key elements of study design early in the paper	8
Setting	5	Describe the setting, locations, and relevant dates, including periods of recruitment, exposure, follow-up, and data collection	8
Participants	6	(*a*) Give the eligibility criteria, and the sources and methods of selection of participants. Describe methods of follow-up	8
		(*b*) For matched studies, give matching criteria and number of exposed and unexposed	
Variables	7	Clearly define all outcomes, exposures, predictors, potential confounders, and effect modifiers. Give diagnostic criteria, if applicable	9,10
Data sources/ measurement	8*	For each variable of interest, give sources of data and details of methods of assessment (measurement). Describe comparability of assessment methods if there is more than one group	9,10
Bias	9	Describe any efforts to address potential sources of bias	11
Study size	10	Explain how the study size was arrived at	9
Quantitative variables	11	Explain how quantitative variables were handled in the analyses. If applicable, describe which groupings were chosen and why	9,10
Statistical methods	12	(*a*) Describe all statistical methods, including those used to control for confounding	11,12
		(*b*) Describe any methods used to examine subgroups and interactions	
		(*c*) Explain how missing data were addressed	
		(*d*) If applicable, explain how loss to follow-up was addressed	
		(*e*) Describe any sensitivity analyses	
**Results**			
Participants	13*	(a) Report numbers of individuals at each stage of study—eg numbers potentially eligible, examined for eligibility, confirmed eligible, included in the study, completing follow-up, and analysed	12
		(b) Give reasons for non-participation at each stage	
		(c) Consider use of a flow diagram	
Descriptive data	14*	(a) Give characteristics of study participants (eg demographic, clinical, social) and information on exposures and potential confounders	12,13, 24
		(b) Indicate number of participants with missing data for each variable of interest	
		(c) Summarise follow-up time (eg, average and total amount)	
Outcome data	15*	Report numbers of outcome events or summary measures over time	12,13

For peer review only - http://bmjopen.bmj.com/site/about/guidelines.xhtml

Main results	16	(*a*) Give unadjusted estimates and, if applicable, confounder-adjusted estimates and their precision (eg, 95% confidence interval). Make clear which confounders were adjusted for and why they were included<br>(*b*) Report category boundaries when continuous variables were categorized<br>(*c*) If relevant, consider translating estimates of relative risk into absolute risk for a meaningful time period	11-13<br>27
Other analyses	17	Report other analyses done—eg analyses of subgroups and interactions, and sensitivity analyses	13,14
**Discussion**			
Key results	18	Summarise key results with reference to study objectives	14,15
Limitations	19	Discuss limitations of the study, taking into account sources of potential bias or imprecision. Discuss both direction and magnitude of any potential bias	16,17
Interpretation	20	Give a cautious overall interpretation of results considering objectives, limitations, multiplicity of analyses, results from similar studies, and other relevant evidence	14-17
Generalisability	21	Discuss the generalisability (external validity) of the study results	16,17
**Other information**			
Funding	22	Give the source of funding and the role of the funders for the present study and, if applicable, for the original study on which the present article is based	Author statement form

*Give information separately for exposed and unexposed groups.

Note: An Explanation and Elaboration article discusses each checklist item and gives methodological background and published examples of transparent reporting. The STROBE checklist is best used in conjunction with this article (freely available on the Web sites of PLoS Medicine at http://www.plosmedicine.org/, Annals of Internal Medicine at http://www.annals.org/, and Epidemiology at http://www.epidem.com/). Information on the STROBE Initiative is available at http://www.strobe-statement.org.

For peer review only

For peer review only - http://bmjopen.bmj.com/site/about/guidelines.xhtml

BMJ Open

Characteristics and management of adolescents attending the ED with fever: a prospective multicentre study.

Journal:	*BMJ Open*
Manuscript ID	bmjopen-2021-053451.R1
Article Type:	Original research
Date Submitted by the Author:	24-Nov-2021

Complete List of Authors:	Borensztajn, Dorine; Erasmus MC Sophia Children Hospital, Department of Pediatrics<br>Hagedoorn, Nienke; Erasmus MC Sophia Children Hospital, Department of Pediatrics<br>Carrol, Enitan; University of Liverpool, Institute of Infection and Global Health<br>von Both, Ulrich; Munich University Hospital Dr von Hauner Children's Hospital<br>Dewez, Juan ; London School of Hygiene and Tropical Medicine, Clinical Research Department<br>Emonts, Marieke; Great North Children's Hospital, Paediatric Immunology, Infectious Diseases & Allergy, Newcastle upon Tyne Hospitals NHS Foundation Trust<br>van der Flier, Michiel; Radboudumc, Pediatric Infectious Diseases and Immunology<br>de Groot, Ronald; Radboudumc, Pediatric Infectious Diseases and Immunology<br>Herberg, Jethro; Imperial College London, Paediatrics<br>Kohlmaier, Benno; Medical University of Graz, Department of General Paediatrics<br>Levin, M; Imperial College London<br>Lim, Emma; Great North Children's Hospital<br>Maconochie, Ian; Imperial College London, Paediatric Emergency Department<br>Martinon Torres, Federico; Complexo Hospitalario Universitario de Santiago de Compostela,<br>Nijman, R; Imperial College Healthcare NHS Trust, Department of Paediatrics<br>Pokorn, Marko; University of Ljubljana, Department of Infectious Diseases<br>Rivero-Calle, Irene; University Hospital of Santiago de Compostela, Pediatrics<br>Tsolia, Maria; National and Kapodistrian University of Athens, Department of Paediatric Infectious Diseases, University of Athens<br>Vermont , Clementien ; Erasmus MC Sophia Children Hospital, Department of paediatric infectious diseases and immunology<br>Zavadska, Dace; Riga Stradins University, Department of Pediatrics<br>Zenz, Werner; Medical University of Graz, Department of General Paediatrics<br>Zachariasse, Joany; Erasmus MC Sophia,<br>moll, Henriette; Erasmus MC Sophia Children Hospital, Department of

	Pediatrics
**Primary Subject Heading**:	Paediatrics
Secondary Subject Heading:	Emergency medicine
Keywords:	PAEDIATRICS, Paediatric infectious disease & immunisation < PAEDIATRICS, Paediatric A&E and ambulatory care < PAEDIATRICS

SCHOLARONE™
Manuscripts

I, the Submitting Author has the right to grant and does grant on behalf of all authors of the Work (as defined in the below author licence), an exclusive licence and/or a non-exclusive licence for contributions from authors who are: i) UK Crown employees; ii) where BMJ has agreed a CC-BY licence shall apply, and/or iii) in accordance with the terms applicable for US Federal Government officers or employees acting as part of their official duties; on a worldwide, perpetual, irrevocable, royalty-free basis to BMJ Publishing Group Ltd ("BMJ") its licensees and where the relevant Journal is co-owned by BMJ to the co-owners of the Journal, to publish the Work in this journal and any other BMJ products and to exploit all rights, as set out in our licence.

The Submitting Author accepts and understands that any supply made under these terms is made by BMJ to the Submitting Author unless you are acting as an employee on behalf of your employer or a postgraduate student of an affiliated institution which is paying any applicable article publishing charge ("APC") for Open Access articles. Where the Submitting Author wishes to make the Work available on an Open Access basis (and intends to pay the relevant APC), the terms of reuse of such Open Access shall be governed by a Creative Commons licence – details of these licences and which Creative Commons licence will apply to this Work are set out in our licence referred to above.

Other than as permitted in any relevant BMJ Author's Self Archiving Policies, I confirm this Work has not been accepted for publication elsewhere, is not being considered for publication elsewhere and does not duplicate material already published. I confirm all authors consent to publication of this Work and authorise the granting of this licence.

For peer review only

Characteristics and management of adolescents attending the ED with fever: a prospective multicentre study.

Dorine M. Borensztajn[1], Nienke N. Hagedoorn[1], Enitan D. Carrol[2,3,4], Ulrich von Both[5,6], Juan Emmanuel Dewez[7], Marieke Emonts[8,9,10], Michiel van der Flier[11,12], Ronald de Groot[11], Jethro Herberg[13], Benno Kohlmaier[14], Michael Levin[13], Emma Lim[8], Ian K. Maconochie[13], Federico Martinón-Torres[15], Ruud G. Nijman[13], Marko Pokorn[16], Irene Rivero-Calle[15], Maria Tsolia[17], Clementien Vermont[18], Dace Zavadska[19], Werner Zenz[14], Joany M. Zachariasse[1], Henriette A. Moll[1]

On behalf of PERFORM consortium: Personalised Risk assessment in febrile children to optimise Real-life Management across the European Union.*

Corresponding author:

Dorine Borensztajn

Department of General Paediatrics, Erasmus MC – Sophia Children's Hospital

P.O. Box 2060, 3000 CB, Rotterdam, the Netherlands

Telephone: +31 10 703 61 96

E-mail: d.borensztajn@erasmusmc.nl

Author affiliations:

1. Erasmus MC-Sophia Children's Hospital, Department of General Paediatrics, Rotterdam, the Netherlands.

For peer review only - http://bmjopen.bmj.com/site/about/guidelines.xhtml

2. University of Liverpool, Institute of Infection, Veterinary and Ecological Sciences, Liverpool, United Kingdom.
3. Alder Hey Children's NHS Foundation Trust, Department of Infectious Diseases, Liverpool, UK
4. Liverpool Health Partners, Liverpool, UK
5. Dr. von Hauner Children's Hospital, University hospital, Ludwig-Maximilians-University (LMU), Division of Paediatric Infectious Diseases, München, Germany.
6. German Centre for Infection Research, DZIF, partner site Munich, Germany
7. London School of Hygiene and Tropical Medicine, Faculty of Tropical and Infectious Disease, London, United Kingdom.
8. Great North Children's Hospital, Paediatric Immunology, Infectious Diseases & Allergy, Newcastle upon Tyne Hospitals NHS Foundation Trust, Newcastle upon Tyne, United Kingdom.
9. Translational and Clinical Research Institute, Newcastle University, Newcastle upon Tyne, United Kingdom
10. NIHR Newcastle Biomedical Research Centre based at Newcastle upon Tyne Hospitals NHS Trust and Newcastle University, Newcastle upon Tyne, United Kingdom
11. Stichting Katholieke Universiteit, Radboudumc Nijmegen, The Netherlands.
12. Wilhelmina Children's Hospital, University Medical Centre Utrecht, Department of Paediatric Infectious Diseases and Immunology, Utrecht, The Netherlands.
13. Imperial College of Science, Technology and Medicine, Section of Paediatric Infectious Diseases, London, United Kingdom.
14. Medical University of Graz, Department of General Paediatrics, Graz, Austria.

For peer review only

For peer review only - http://bmjopen.bmj.com/site/about/guidelines.xhtml

15. Hospital Clínico Universitario de Santiago de Compostela, Genetics, Vaccines, Infections and Pediatrics Research group (GENVIP), Santiago de Compostela, Spain
16. University Medical Centre Ljubljana, Univerzitetni Klinični Center, Department of Infectious Diseases, Ljubljana, Slovenia.
17. National and Kapodistrian University of Athens, Second Department of Paediatrics, P. and A. Kyriakou Children's Hospital, Second Department of Paediatrics, Athens, Greece.
18. Erasmus MC-Sophia Children's Hospital, Department of Pediatric infectious diseases & immunology, Rotterdam, the Netherlands.
19. Rīgas Stradiņa universitāte, Children clinical university hospital, Department of Pediatrics, Riga, Latvia

*Membership of the PERFORM Consortium is provided in appendix 1.

For peer review only

Abbreviations

aOR – adjusted odds ratio

CRP – C-reactive protein

ED – Emergency Department

EMS – Emergency Medical Services

ILSI – Immediate life-saving interventions

MOFICHE – Management and Outcome of Fever in children in Europe

NICE - National Institute for Health and Care Excellence

OR – odds ratio

For peer review only - http://bmjopen.bmj.com/site/about/guidelines.xhtml

PERFORM - Personalized Risk assessment in Febrile illness to Optimize Real-life Management across the European Union

PCR – Polymerase Chain Reaction

PEWS - Paediatric Early Warning Score

PICU – Paediatric Intensive Care Unit

SBI – Serious bacterial infections

Word count: 3229

Funding:

This project has received funding from the European Union's Horizon 2020 research and innovation programme under grant agreement No. 668303

The Research was supported by the National Institute for Health Research Biomedical Research Centre based at Imperial College (JH, ML) and at Newcastle Hospitals NHS Foundation Trust and Newcastle University (EL, ME), grant number not applicable.

The views expressed are those of the author(s) and not necessarily those of the NHS, the NIHR or the Department of Health.

Contributors:

Conceptualisation and design: DB, NH, EC, UB, JD, ME, MF, RG, JH, BK, ML, EL, IM, FT, RN, MP, IR, MT, DZ, WZ, HM

Data acquisition: DB, NH, EC, UB, JD, ME, MF, RG, JH, BK, ML, EL, IM, FT, RN, MP, IR, MT, CV, DZ, WZ, JZ, HM

Data verification: DB, NH

Data interpretation: DB, NH, HM

Formal analysis: DB, NH

Writing—original manuscript: DB

For peer review only

Critically revising the manuscript: DB, NH, EC, UB, JD, ME, MF, RG, JH, BK, ML, EL, IM, FT, RN, MP, IR, MT, CV, DZ, WZ, JZ, HM

The corresponding author attests that all listed authors meet authorship criteria and that no others meeting the criteria have been omitted. This publication is the work of the authors who will serve as guarantors for the contents of this paper.

Competing interest:

The authors declare they have no potential conflicts of interest to disclose.

For peer review only

Abstract

Objective

Most studies regarding febrile children have focused on infants and young children with serious bacterial infections (SBI). Although population studies have described an increased risk of sepsis in adolescents, little is known about febrile adolescents attending the Emergency Department (ED). We aimed to describe patient characteristics and management of febrile adolescents attending the ED.

Design and setting

The MOFICHE/PERFORM, a prospective multicentre study, took place at 12 European EDs. Descriptive and multivariable regression analysis were performed, comparing febrile adolescents (12-18 years) to younger children in terms of patient characteristics, markers of disease severity (vital signs, clinical alarming signs), management (diagnostic tests, therapy, admission) and diagnosis (focus, viral/bacterial infection).

Results

37,420 encounters were included, of which 2,577 (6·9%) were adolescents.

Adolescents were more often triaged as high-urgent (38·9% versus 34·5%) and described as ill-appearing (23·1% versus 15·6%) than younger children.

Increased work of breathing and a non-blanching rash were present less often in adolescents while neurological signs were present more often (1% versus 0%). C-reactive protein was performed more frequently in adolescents and was abnormal more often (aOR 1·7, 95%CI 1·5-1·9). Adolescents were more often diagnosed with SBI (OR 1·8, 95%CI 1·6-2·0) and sepsis/meningitis (OR 2·3, 95%CI 1·1-5·0), more frequently were admitted (aOR 1·3, 95% CI 1·2-1·4) and treated with intravenous antibiotics (aOR 1·7, 95% CI 1·5-2·0).

For peer review only - http://bmjopen.bmj.com/site/about/guidelines.xhtml

Conclusions

Although younger children presented to the ED more frequently, adolescents were more often diagnosed with SBI and sepsis/meningitis. Our data emphasize the importance of awareness for severe infections in adolescents.

Keywords

Adolescent health, epidemiology.

Article summary (strengths and limitations of this study)

- Our study provides detailed data on a large number of adolescents attending the Emergency Department with fever
- Our data show that although accounting for a relatively small fraction of all Emergency Department visits for febrile children, adolescents have an increased risk of serious bacterial infections
- Febrile adolescents are hospitalized more often, are treated with intravenous antibiotics more often and more often required immediate life-saving interventions
- Adolescents with serious bacterial infections present differently than younger children and more research is needed to be able to provide detailed guidelines for this age group

For peer review only

For peer review only - http://bmjopen.bmj.com/site/about/guidelines.xhtml

INTRODUCTION

The adolescent age is often described as a health paradox because on the one hand it is a time of enhanced physical and mental capabilities, yet the overall mortality/morbidity rates increase significantly, often due to risk taking behaviour such as substance abuse or injuries.[(1)]

Although this health paradox has received considerable attention in the international literature, there is a knowledge gap regarding infectious problems in adolescents, as most studies on adolescents have focused on topics typically associated with adolescence, such as violence or mental health,[(2) (3) (4)] while most studies on infections have focused on younger children.[(5)]

Case series as well as population studies have shown adolescents to have an increased risk of sepsis in comparison to younger children[(6, 7)] and several studies showed adolescents with sepsis to have an increased mortality rate. [(6–11)]

One possible explanation of this increased case-fatality rate might be the atypical presentation of adolescents with sepsis, such as gastro-intestinal complaints. [(9, 12)] The increased incidence of sepsis and the high case-fatality rates emphasize the importance of awareness for severe infections in adolescents.

Despite this, little is known on the presentation, management and diagnosis of febrile adolescents presenting to the Emergency Department (ED). Our aim was to assess the presentation, management and diagnosis of febrile adolescents attending the ED and explore differences between adolescents and younger children.

For peer review only - http://bmjopen.bmj.com/site/about/guidelines.xhtml

METHODS

Study design

This study is part of the MOFICHE study (Management and Outcome of Febrile children in Europe), which is embedded in the PERFORM study (Personalized Risk assessment in Febrile illness to Optimise Real-life Management across the European Union).[13] MOFICHE is an observational multicentre study that evaluates the management and outcome of febrile children in Europe using routinely collected data.[14] In this sub-study we specifically assessed patient characteristics, diagnosis and management of febrile adolescents and compared it to the characteristics and management of younger children.

Patient and Public Involvement

Patients were not involved in the design of the study.

Study population and setting

Twelve EDs from eight different countries (Austria, Germany, Greece, Latvia, the Netherlands (n=3), Spain, Slovenia, and the United Kingdom (n=3)) participated in the study (appendix 1). Participating hospitals were either tertiary university hospitals or large teaching hospitals (appendix 2). Data were collected for at least one year (January 2017-April 2018).

For this analysis, inclusion criteria were children aged 3 months-18 years presenting to the ED with fever (temperature >=38·0° C) or a history of fever in the previous 72 hours.

Data collection

Data were obtained from patient records and entered into an electronic case report form. Data included general patient characteristics (age, sex, comorbidity, previous medical care, arrival time, referral (self, primary care physician, Emergency Medical Services (EMS) or

For peer review only - http://bmjopen.bmj.com/site/about/guidelines.xhtml

other), triage urgency, vital signs, presence of “red traffic light” alarming signs from the National Institute for Health and Care Excellence (NICE) fever guideline,[15] high risk criteria from the NICE sepsis guideline[15] (table 1) and management at the ED. The NICE alarming signs include level of consciousness, ill-appearance, increased work of breathing, age <3 months, non-blanching rash, meningeal signs, status epilepticus and focal neurological signs. High risk criteria from the NICE Sepsis guideline overlap with NICE alarming signs but differ by age-group and include abnormal behaviour, decreased consciousness, low oxygen saturation, abnormal heart rate, respiratory rate or blood pressure, hypothermia, age <3 months, diminished urine output, cyanosis or a non-blanching rash. An overview of the collected alarming signs is provided in table 2.

Data collection ranged from one week per month to the entire month, depending on the number of ED visits per hospital (appendix 2).

Management comprised diagnostic tests (performance of blood tests, imaging, blood cultures and CRP test results), treatment (intravenous antibiotics, oxygen, Immediate Life Saving Interventions (ILSI)) and disposition (discharge, general ward admission or Paediatric Intensive Care unit (PICU) admission).

Definitions

Adolescents were defined as children aged 12-18 years; younger children were defined as children aged 3 months-12 years.

Previous medical care was defined as medical care for the same complaint in the last 5 days at any facility, including a general practitioner. A previous ED visit was defined as a visit to either the same or a different ED in the previous five days. Comorbidity was defined as a chronic underlying condition that is expected to last at least 1 year.[16]

For peer review only - http://bmjopen.bmj.com/site/about/guidelines.xhtml

Vital signs were classified as abnormal according to Advanced Paediatric Life Support (APLS) reference ranges.

Simplified Paediatric Early Warning Scores (PEWS) were calculated based on the PEWS developed by Parshuram et al. (vital signs, capillary refill time, work of breathing and oxygen therapy, combined into a score).[17, 18] Blood pressure was excluded from the PEWS as it was not routinely performed in our study. A previous study showed that a simplified PEWS without blood pressure showed similar performance in predicting PICU admission in comparison to the original PEWS.[18]

Triage categories were combined into “low urgency” (non-urgent and standard) and “high urgency” (urgent, very urgent and immediate).

ILSI[19] were categorized into the following categories: airway/breathing support, electrical therapy, emergency procedures, hemodynamic support and emergency medications (appendix 3).

Focus of infection was categorized as: upper respiratory, lower respiratory, gastro-intestinal/surgical abdomen, urinary, skin, musculoskeletal, sepsis, meningitis/central nervous system (CNS), flu-like illness, childhood exanthemas, inflammatory, undifferentiated fever or other.[14]

The consortium developed a consensus-based flowchart [14] [20] [21] to classify the presumed cause of infection for each visit (appendix 4), depending on clinical signs, C-reactive protein (CRP) and microbiological tests (bacterial cultures, viral or bacterial polymerase chain reaction (PCR)), into “definite or probable bacterial”, “definite or probable viral”, “unknown” or “other”.

Serious bacterial infections (SBI) were defined as ‘definite/probable bacterial’ with a focus from the gastro-intestinal, lower respiratory, urinary or musculoskeletal tract, CNS or sepsis.

For peer review only - http://bmjopen.bmj.com/site/about/guidelines.xhtml

Sepsis/meningitis was defined as 'definite/probable bacterial' with a focus from the CNS or sepsis.

Data quality and missing data

Data quality and completeness was improved and standardized by using a digital training module for physicians who assess febrile children at the ED, including clarification of the NICE alarming signs. Data were entered into the patient's record as part of routine care by the treating physician and nurse and were then manually extracted from these records and entered into an electronic case report form by trained research team members.

Missing determinants such as vital signs were handled by multiple imputation (table 1). Imputation was performed by using the MICE package in R, version 3.4. SPSS version 25 was used for the analysis of the data.

Data analysis

We performed descriptive analyses for general patient characteristics, vital signs, NICE alarming signs, management, disposition and diagnosis. Characteristics of adolescents and younger children were compared using chi-squared-tests and Mann-Whitney tests. Results were deemed significant with a p-value $<0{\cdot}05$.

We analysed differences in management, disposition and presumed cause of infection by multivariable logistic regression, displayed as odds ratios (OR), and adjusted for general patient characteristics (setting/ED, sex, fever duration, previous medical care, arrival time and comorbidity), displayed as adjusted odds ratios (aOR). We did not adjust for disease severity as our aim was to describe differences in disease severity between young children and

For peer review only - http://bmjopen.bmj.com/site/about/guidelines.xhtml

adolescents. Subgroup analyses were performed for children and adolescents diagnosed with SBI and for children without comorbidity.

RESULTS

Patient characteristics

37,420 ED encounters were included, of which 2,577 (6·9%) were adolescents (table 1). Adolescents were less often self-referred (49·4% versus 57·8%) and more often presented by EMS than younger children (17·3% versus 14·8%, p< 0·001). 2,816 (8·1%) younger children and 239 adolescents (9·3%) had attended an ED in the previous five days. Adolescents more often had comorbidity (28·5% versus 16·4%, p<0·001, table 1, appendix 5).

Presenting signs and symptoms

Adolescents were more often triaged as high urgent. Tachycardia was present more often (29·6% versus 24·5%, p<0·001) while tachypnoea, increased work of breathing and low oxygen saturation were present less often.

Adolescents more often had a PEWS score of 6 or higher (6·2% versus 4·5%, p<0·001).

Non-blanching rashes were present less often in adolescents while ill-appearance, meningeal signs and focal neurological signs were present more often (table 1).

In a sub-analysis of children without comorbidity, results were similar, except triage urgency, PEWS and focal neurological signs, which were then similar in both groups.

For peer review only - http://bmjopen.bmj.com/site/about/guidelines.xhtml

Management

After adjusting for general patient characteristics, we found that diagnostic tests were such as CRP were performed more often in adolescents (aOR 1·9, 95% CI 1·7-2·0) and CRP more often reached levels >60 mg/l (aOR 1·7, 95% CI 1·5-1·9).

Hospital admission (aOR 1·3, 95% CI 1·2-1·4), intravenous antibiotics (aOR 1·7, 95% CI 1·5-1·9) and ILSI (aOR 1·5, 95% 1·2-2·0) were more common in adolescents while PICU admission was similar in both age-groups (aOR 1·1, 95% CI 0·6-1·9, figure 1, figure 2).

Of those children that had attended the ED previously, 36·1% of younger children and 49·0% of adolescents were admitted (p<0·001). ICU admission was similar for younger children (0·9%) and adolescents (0·8%).

Sub-analysis in children without comorbidity showed similar results, except for ILSI, which was similar in both groups (figure 3, figure 4).

Focus and presumed cause of infection

Upper respiratory tract infection was the most common focus in both age-groups, although this was less common in adolescents than in younger children (41·8% versus 53·9%, figure 5, p<0·001). Gastro-intestinal/surgical abdomen was diagnosed more often in adolescents (16·2%) (10·1%, p<0·001). Adolescents were more often classified as having bacterial disease (31·0% versus 21·6%, aOR 1·5, 95% CI 1·4-1·7) and SBI (15·8% versus 8·4%, aOR 1·8, 95%CI 1·6-2·0), and less often with probable/definite viral disease. Most common SBI in both adolescents and younger children were lower respiratory tract, urinary tract and gastro-intestinal infections. Bacterial sepsis/meningitis was more common in adolescents (0·6% versus 0·3%, OR 1·9 (1·1-3·3)), although after adjusting for general patient characteristics, this was significant only in children without comorbidity (aOR 2·3, 95% CI 1·1-5·0, figure 4).

For peer review only - http://bmjopen.bmj.com/site/about/guidelines.xhtml

Of those children that had attended the ED previously, 13·8% of younger children and 21·8 of adolescents were diagnosed with SBI (p<0·001).

Presentation and management of children and adolescents with SBI.

In total 3,347 children presented with SBI. SBI were present in 406 of 2,577 adolescents (15·8%) and 2,941 of 34,843 of younger children (8·4%).

Adolescents with SBI more often had comorbidity (34·0% versus 23·6%, p<0·001), less often presented with tachypnoea and increased work of breathing, while rates of tachycardia and prolonged capillary refill were similar between adolescents and younger children with SBI. Adolescents with SBI and sepsis/meningitis were more often described as ill-appearing and adolescents that were described as ill-appearing, more often were diagnosed with SBI or sepsis/meningitis. However, high-risk criteria from the NICE sepsis guideline were present less frequently in adolescents with SBI (appendix 6).

No differences were found regarding the frequency of CRP >60mg/l, intravenous antibiotics, admission or PICU admission. Adolescents with SBI more often were treated with ILSI than younger children (aOR 2·2, 95% CI 1·4-3·5), although this difference was not significant after excluding children with comorbidity.

DISCUSSION

Main findings

A well-known-statement emphasizes how "children are not small adults", [22] [23] however, our data show that adolescents are not large children either.

Our data show that despite accounting for a small fraction of all ED visits for febrile children,

For peer review only - http://bmjopen.bmj.com/site/about/guidelines.xhtml
For peer review only

adolescents presenting to the ED have an increased risk of SBI such as sepsis/meningitis. Furthermore, adolescents with SBI present differently than younger children. Although adolescents were more often described as ill-appearing, high-risk criteria from the NICE sepsis guideline were present less frequently in adolescents with SBI or sepsis/meningitis. Adolescents were hospitalized more often, and more often received intravenous antibiotics and ILSI.

Findings in relation to previous literature

Previous studies on febrile children have mainly focused on infants and young children, [5] and literature regarding febrile adolescents is scarce. A recent study by Brockhus et al. on adolescents attending the ED, showed that adolescents present with complaints different from those in children as well as adults and infectious problems were far less common than trauma or mental health issues. [23]

However, as stated above, although adolescents present with infectious problems less common than younger children, adolescents that *do* present to the ED, have an increased risk of suffering from a severe infection. Our data is in line with the few studies that have found an increased incidence of sepsis in adolescents in comparison to younger children [7] [9] and emphasize how febrile adolescents form a distinct risk group. In line with this, a study by Glynn et al [24] showed the severity of several infectious diseases (e.g. varicella, mononucleosis, meningococcal infections and scarlet fever) to be high in infancy, lower in school-aged children and then again increasing from the adolescent age, following a J-shaped pattern.

To date it is unclear why adolescents have an increased risk for serious infections. Possible explanations include immunological deterioration with age, so-called “immune senescence”, the influence of sex hormones on the immune system or the increase of comorbidity with age. [24] Regarding immune senescence, Glynn et al suggest that this process starts earlier

For peer review only - http://bmjopen.bmj.com/site/about/guidelines.xhtml

than previously believed, with optimal immune function being reached at age 5-14, and a decrease in immune function starting form adolescence. [24] Evidence supporting this theory comes from data on vaccine response by age, showing a decreased vaccine response in adolescents. [24] Regarding puberty and hormonal influences, data seems to be inconsistent as the increased mortality rates in infectious diseases seen in males in comparison to females is not seen in adolescence. [24] Regarding comorbidity, although in our study adolescents more often had comorbidity, SBI were still more common in adolescents after excluding children with comorbidity and sepsis/meningitis was more common in adolescents only in the subgroup of children without comorbidity, showing that comorbidity does not offer a clear-cut explanation for these trends.

In addition to presenting with different rates of the same diseases, adolescents with the same disease can present differently as well. [12] As stated before, although not "small adults", adolescents are not "large children" either, differing from both adults and children with regards to physiology, the immune system and the endocrine system. [25] Further differences might be explained by differences in health seeking behavior, lack of parental supervision or a delayed presentation [12] [10] [26] although the latter was not the case in our study. Regarding parental supervision, this was shown to be related to treatment adherence in adolescents with cystic fibrosis [27] or diabetes, [28] but there is a paucity of data regarding parental supervision in adolescents with infectious diseases and its impact on presentation or disease course.

For peer review only

Our data show that the *health paradox* does not only apply to preventable injuries, but also to potentially vaccine-preventable infections, such as meningococcal disease, or treatable infectious diseases, such as sepsis, where early recognition has the potential to improve outcome. [10] [29]

Implications for clinical practice and research

Our data highlight a gap in clinical guidelines addressing the presentation and management of febrile adolescents. While the NICE sepsis guideline addresses adolescents as well as younger children, [15] its focus is on the recognition of sepsis, while the more general NICE fever guideline is exclusively targeted at children below the age of five and our data show how the alarming signs for this age group cannot be extrapolated unambiguously to adolescents. [15] Furthermore, as most studies on adolescents focus on mental health issues, there's a paucity of literature on febrile adolescents.

As adolescents with infectious problems form only a small fraction of ED visits [23] exposure for each individual health care provider is expected to be low, [30] making the management of this group even more challenging. Therefore, there's an urgent need for future studies directed at identifying clinical criteria that can help improve the identification of SBI in the febrile adolescent age group.

While awaiting future studies, health care workers evaluating adolescents should have an increased level of awareness regarding the substantial risk in adolescents for SBI and the potentially different presentation.

Diagnostic tests and antibiotic therapy should be considered at a low threshold when SBI cannot be ruled out on clinical grounds. In addition to ordering routine tests such as C-reactive protein and white blood count, clinicians should consider performing additional

For peer review only - http://bmjopen.bmj.com/site/about/guidelines.xhtml

tests such as lactate and procalcitonin, as these offer improved diagnostic performance when it comes to differentiating sepsis from other causes of fever. [31] [32] [33] [34] [15]

Secondly, safety netting advice should be given to all febrile adolescents and their caregivers in case of discharge from the ED. Empowering adolescents on when to seek help and when and how to self-care at home is an important step in the management of febrile illnesses in adolescents. Most studies regarding patient's knowledge on fever have focused on caregivers of young children, [35] [36] as do many online information sources.[37] Previous research on how to improve empowerment in adolescents with medical problems can aid in optimally addressing this specific population as this requires a different approach than addressing adults or parents of younger children. [38] [39]

STRENGTHS AND LIMITATIONS

To our knowledge this is the first study looking into patient characteristics, management and diagnosis of febrile adolescents attending the ED.

The main strengths of our study are that detailed information on presenting signs, management and diagnosis was collected on a large number of children and adolescents in different European EDs. Data were collected year-round and included different hospitals with different patient case mixes, which largely increases the generalizability of the results.[14] [40] Furthermore, we included a large number of children with SBI, as determined by a uniformly applied flowchart.

The main limitations include the lack of information regarding outcome after the ED visit, e.g. 30-day morbidity and mortality in the use of routinely collected data. To ensure data quality, all study sites were extensively trained regarding the accurate documentation of

For peer review only - http://bmjopen.bmj.com/site/about/guidelines.xhtml

patient characteristics and quality checks were performed regularly. The amount of missing data was limited and its effects were reduced by using multiple imputation.[41] Another limitation is that blood pressure, cyanosis and diminished urine output were not included in the data collection. A previous study showed that although hypotension is associated with serious illness in children, its sensitivity is limited as routine measurement in *all* children attending the ED. [42, 43] and it is a late sign in septic children in comparison to adults. On the other hand, in adolescents, similar to adults, hypotension might present earlier in the disease course, and thus including blood pressure could provide valuable information.

As cases defined as 'probable bacterial' were also included in the definition of sepsis/meningitis, we cannot preclude that some of these cases were not of bacterial origin in either age-groups. However, in the EUCLIDS study on severe sepsis, a pathogen was only found in half of the cases.[11] Lastly, our data applies to adolescents attending the ED; more research is needed to know whether our results can be applied to adolescents presenting to primary care as well. Furthermore, it is unknown whether a form of "selection bias" exists, as parents might be more inclined to seek help for younger febrile children than for adolescents.

CONCLUSION

Our data show that despite accounting for a relatively small fraction of all ED visits, febrile adolescents have an increased risk of serious bacterial infections including sepsis/meningitis, in comparison to younger children.

For peer review only - http://bmjopen.bmj.com/site/about/guidelines.xhtml

For peer review only

For peer review only - http://bmjopen.bmj.com/site/about/guidelines.xhtml

Data availability

Individual participant data that underlie the results reported in this article, including a data dictionary, will be made available after de-identification to researchers who provide a methodologically sound proposal. Proposals should be directed to d.borensztajn@erasmusmc.nl. To gain access, data requestors will need to sign a data access agreement.

For peer review only

Tables and figures

For peer review only - http://bmjopen.bmj.com/site/about/guidelines.xhtml

Table 1: Differences in patient characteristics between young children and adolescents (n=37,420)

	Children 3 months- 12 years N = 34,843 N (%)	Children ≥ 12 years N = 2,577 N (%)
**Male**	19,182 (55·1)	1,307 (50·7)
**Age in years, median (IQR)**	2·6 (1·3-4·9)	14·5 (13·2-16·1)
**Comorbidity**[a]		
**Simple**	4,302 (12·5)	489 (19·1)
**Complex**	1,332 (3·9)	241 (9·4)
**Duration of fever** *		
**< 24 hours**	11,410 (35·1)	854 (37·3)
**24-48 hours**	10,622 (32·7)	682 (29·8)
**> 48 hours**	10,433 (31·1)	755 (33·0)
**Referral**		
**Self**	19,537 (57·8)	1,231 (49·4)
**GP/private paediatrician**	5,654 (16·7)	493 (19·8)
**Emergency medical service**	5,010 (14·8)	430 (17·3)
**Other**	3,574 (10·6)	337 (13·5)
**Triage urgency**		
**High: immediate, very urgent, intermediate**	11,664 (34·5)	967 (38·9)
**Vital signs**[b] **and PEWS**		
**Tachycardia APLS**	8,552 (24·5)	764 (29·6)
**Tachypnoea APLS**	5,282 (15·2)	189 (7)
**Hypoxia, oxygen saturation <95% APLS**	805 (2·3)	30 (1)
**Prolonged capillary refill >=3 seconds**[ns]	343 (1·1)	25 (1)
**Simplified PEWS 6 or higher**	782 (4·5)	81 (6)
**Nice "red traffic lights" (alarming signs)**		
**Ill appearance**	5,203 (15·6)	559 (23·1)
**Increased work of breathing**	3,050 (10·0)	67 (3)
**Rash: petechiae/non-blanching**	1,040 (3·4)	53 (2)
**Decreased consciousness**[ns]	178 (1)	16 (1)
**Meningeal signs**	97 (0)	23 (1)
**Status epilepticus**[ns]	58 (0)	8 (0)
**Focal neurology**	110 (0)	19 (1)

Missing values: general patient characteristics: < 7%. Vital signs: 9-23%. NICE alarming signs 1-18%.
[a]. Comorbidity: a chronic underlying condition that is expected to last at least 1 year. Complex comorbidity: a chronic condition in ≥2 body systems or malignancy or immunocompromised patients.
[b]. According to APLS cut-off values by age.
All comparisons p<0·001, except * = <0·05 and [ns] = not significant.

For peer review only

Table 2. "Red traffic light" symptoms (alarming signs) from the NICE guideline on fever and high-risk criteria from the NICE sepsis guideline.

	Fever < 5[a]	Sepsis < 5[b]	Sepsis 5-11[b]	Sepsis ≥12[b]
**Behaviour**				
No response to social cues[c]	+	+		
Altered behaviour[c]			+	+
Ill appearance	+	+	+	
Does not wake / does not stay awake	+	+	+	
Weak high-pitched or continuous cry[d]	+	+		
**Respiratory**				
Grunting	+	+		
Apnoea		+		
Oxygen saturation < 90%		+	+	
Oxygen saturation < 93%				+
Tachypnoea for age	+	+	+	+
Chest retractions	+			
**Circulation**				
Bradycardia < 60		+	+	
Tachycardia for age		+	+	+
Reduced skin turgor	+			
Not passed urine in previous 18 hours.				+
Systolic blood pressure 90 mmHg				+
**Skin**				
Mottled, ashen or cyanosis	+	+	+	+
Non-blanching rash	+	+	+	+
**Temperature**				
< 36·0º C		+		
≥38·0 in infants < 3 months	+	+		
**Neurological**				
Bulging fontanelle or neck stiffness	+			
Status epilepticus	+			
Focal neurological signs	+			
Focal seizures	+			
		Data available	Available (proxy used)	Not available

[a] NICE fever guideline
[b] NICE sepsis guideline
[c] Defined as reduced consciousness
[d] defined as ill appearance

For peer review only - http://bmjopen.bmj.com/site/about/guidelines.xhtml

Figure 1: Adjusted odds ratios for younger children versus adolescents for diagnostic tests and therapy. [a]

[a] Younger children used as reference. Adjusted for hospital, sex, duration of fever, previous medical care, time of arrival and comorbidity.

To convert CRP values to nmol/L, multiply by 0·9524

Figure 2: Adjusted odds ratios for younger children versus adolescents for disposition and final diagnosis.[a]

[a] Younger children used as reference. Adjusted for hospital, sex, duration of fever, previous medical care, time of arrival and comorbidity.

Figure 3: Adjusted odds ratios for younger children versus adolescents for diagnostic tests and therapy, patients with comorbidity excluded.[a]

[a] Younger children used as reference. Adjusted for hospital, sex, duration of fever, previous medical care and time of arrival.

To convert CRP values to nmol/L, multiply by 0·9524

Figure 4: Adjusted odds ratios for younger children versus adolescents for disposition and final diagnosis, patients with comorbidity excluded.[a]

[a] Younger children used as reference. Adjusted for hospital, sex, duration of fever, previous medical care and time of arrival.

Figure 5: focus of infection in young children and adolescents

Data shown as percentages within the groups of young children and adolescents.
LRTI=lower respiratory tract infection; gastro-intestinal=gastro-intestinal and surgical abdomen; UTI=urinary tract infection, exanthems=exanthems and flulike illness; musculoskeletal=soft-tissue, skin and musculoskeletal infection.
LRTI (not shown in graphic) = lower respiratory tract infection: young children 54%, adolescents 42%.

For peer review only

Acknowledgments

We gratefully acknowledge the emergency department staff of the participating hospitals for their participation and collection of data. In addition, we thank Arianne van Rijn, (former) medical student for her assistance with the literature review.

Ethics statement

The study was approved by the ethical committees of all the participating hospitals and no informed consent was needed for this study. Austria (Ethikkommission Medizinische Universitat Graz, ID:28-518ex15/16), Germany (Ethikkommission Bei Der LMU München, ID:699-16), Greece (Ethics committee, ID:9683/18.07.2016), Latvia (Centrala medicinas etikas komiteja, ID:14.07.201.6.No. Il16-07-14), Slovenia (Republic of Slovenia National Medical Ethics Committee, ID:0120-483/2016-3), Spain (Comité Autonómico de Ética de la Investigación de Galicia, ID:2016/331), The Netherlands (Commissie Mensgebonden onderzoek, ID:NL58103.091.16), United Kingdom (Ethics Committee, ID:16/LO/1684, IRAS application no. 209035, Confidentiality advisory group reference: 16/CAG/0136).

In all the participating UK settings, an additional opt-out mechanism was in place.

References

1. Willoughby T, Good M, Adachi PJ, Hamza C, Tavernier R. Examining the link between adolescent brain development and risk taking from a social-developmental perspective. Brain Cogn. 2013;83:315-323.
2. Bell TM, Qiao N, Jenkins PC, Siedlecki CB, Fecher AM. Trends in Emergency Department Visits for Nonfatal Violence-Related Injuries Among Adolescents in the United States, 2009-2013. J Adolesc Health. 2016;58:573-575.

For peer review only - http://bmjopen.bmj.com/site/about/guidelines.xhtml

3. Lieberman A, Badolato GM, Tran J, Goyal MK. Frequency of Prescription Filling Among Adolescents Prescribed Treatment for Sexually Transmitted Infections in the Emergency Department. JAMA Pediatr. 2019;173:695-697.
4. Lee J, Bang YS, Min S et al. Characteristics of adolescents who visit the emergency department following suicide attempts: comparison study between adolescents and adults. BMC Psychiatry. 2019;19:231.
5. Cioffredi LA, Jhaveri R. Evaluation and Management of Febrile Children: A Review. JAMA Pediatr. 2016;170:794-800.
6. Angus DC, Linde-Zwirble WT, Lidicker J, Clermont G, Carcillo J, Pinsky MR. Epidemiology of severe sepsis in the United States: analysis of incidence, outcome, and associated costs of care. Crit Care Med. 2001;29:1303-1310.
7. Watson RS, Carcillo JA, Linde-Zwirble WT, Clermont G, Lidicker J, Angus DC. The epidemiology of severe sepsis in children in the United States. Am J Respir Crit Care Med. 2003;167:695-701.
8. Davis KL, Bell TJ, Miller JM, Misurski DA, Bapat B. Hospital costs, length of stay and mortality associated with childhood, adolescent and young adult meningococcal disease in the US. Appl Health Econ Health Policy. 2011;9:197-207.
9. Campbell H, Parikh SR, Borrow R, Kaczmarski E, Ramsay ME, Ladhani SN. Presentation with gastrointestinal symptoms and high case fatality associated with group W meningococcal disease (MenW) in teenagers, England, July 2015 to January 2016. Euro Surveill. 2016;21
10. Burman C, Serra L, Nuttens C, Presa J, Balmer P, York L. Meningococcal disease in adolescents and young adults: a review of the rationale for prevention through vaccination. Hum Vaccin Immunother. 2019;15:459-469.
11. Boeddha NP, Schlapbach LJ, Driessen GJ et al. Mortality and morbidity in community-acquired sepsis in European pediatric intensive care units: a prospective cohort study from the European Childhood Life-threatening Infectious Disease Study (EUCLIDS). Crit Care. 2018;22:143.
12. Thompson MJ, Ninis N, Perera R et al. Clinical recognition of meningococcal disease in children and adolescents. Lancet. 2006;367:397-403.
13. Perform website. Available from: https://www.perform2020.org/.
14. Hagedoorn NN, Borensztajn DM, Nijman R et al. Variation in antibiotic prescription rates in febrile children presenting to emergency departments across Europe (MOFICHE): A multicentre observational study. PLoS Med. 2020;17:e1003208.
15. NICE Guideline. Available from: https://www.nice.org.uk.
16. Simon TD, Cawthon ML, Stanford S et al. Pediatric medical complexity algorithm: a new method to stratify children by medical complexity. Pediatrics. 2014;133:e1647-54.
17. Parshuram CS, Hutchison J, Middaugh K. Development and initial validation of the Bedside Paediatric Early Warning System score. Crit Care. 2009;13:R135.
18. Vredebregt SJ, Moll HA, Smit FJ, Verhoeven JJ. Recognizing critically ill children with a modified pediatric early warning score at the emergency department, a feasibility study. Eur J Pediatr. 2019;178:229-234.
19. Lee JY, Oh SH, Peck EH et al. The validity of the Canadian Triage and Acuity Scale in predicting resource utilization and the need for immediate life-saving interventions in elderly emergency department patients. Scand J Trauma Resusc Emerg Med. 2011;19:68.
20. Herberg JA, Kaforou M, Wright VJ et al. Diagnostic Test Accuracy of a 2-Transcript Host RNA Signature for Discriminating Bacterial vs Viral Infection in Febrile Children. JAMA. 2016;316:835-845.

For peer review only - http://bmjopen.bmj.com/site/about/guidelines.xhtml

21. Martinón-Torres F, Salas A, Rivero-Calle I et al. Life-threatening infections in children in Europe (the EUCLIDS Project): a prospective cohort study. Lancet Child Adolesc Health. 2018;2:404-414.
22. Schramme T, Edwards S. Handbook of the Philosophy of Medicine. Springer; 2017:1100.
23. Brockhus LA, Bärtsch M, Exadaktylos AK, Keitel K, Klukowska-Rötzler J, Müller M. Clinical Presentations of Adolescents Aged 16-18 Years in the Adult Emergency Department. Int J Environ Res Public Health. 2021;18:9578.
24. Glynn JR, Moss PAH. Systematic analysis of infectious disease outcomes by age shows lowest severity in school-age children. Sci Data. 2020;7:329.
25. WHO. Available from: https://www.who.int/ceh/capacity/Children_are_not_little_adults.pdf.
26. Trivedi M, Denton E. Asthma in Children and Adults-What Are the Differences and What Can They Tell us About Asthma. Front Pediatr. 2019;7:256.
27. Modi AC, Marciel KK, Slater SK, Drotar D, Quittner AL. The Influence of Parental Supervision on Medical Adherence in Adolescents With Cystic Fibrosis: Developmental Shifts From Pre to Late Adolescence. Children's Health Care. 2008;37:78-92.
28. Ellis DA, Podolski CL, Frey M, Naar-King S, Wang B, Moltz K. The role of parental monitoring in adolescent health outcomes: impact on regimen adherence in youth with type 1 diabetes. J Pediatr Psychol. 2007;32:907-917.
29. Hilarius KWE, Skippen PW, Kissoon N. Early Recognition and Emergency Treatment of Sepsis and Septic Shock in Children. Pediatr Emerg Care. 2020;36:101-106.
30. Evans IVR, Watson RS, Carcillo J, Angus DC, Seymour CW. Epidemiology of Sepsis Among Adolescents at Community Hospital Emergency Departments: Implications for Rory's Regulations. JAMA Pediatr. 2017;171:1011-1012.
31. Bell JM, Shields MD, Agus A et al. Clinical and Cost-Effectiveness of Procalcitonin Test for Prodromal Meningococcal Disease-A Meta-Analysis. PLoS One. 2015;10:e0128993.
32. Trippella G, Galli L, De Martino M, Lisi C, Chiappini E. Procalcitonin performance in detecting serious and invasive bacterial infections in children with fever without apparent source: a systematic review and meta-analysis. Expert Rev Anti Infect Ther. 2017;15:1041-1057.
33. Hubert-Dibon G, Danjou L, Feildel-Fournial C et al. Procalcitonin and C-reactive protein may help to detect invasive bacterial infections in children who have fever without source. Acta Paediatr. 2018;107:1262-1269.
34. Karon BS, Tolan NV, Wockenfus AM et al. Evaluation of lactate, white blood cell count, neutrophil count, procalcitonin and immature granulocyte count as biomarkers for sepsis in emergency department patients. Clin Biochem. 2017;50:956-958.
35. van de Maat JS, van Klink D, den Hartogh-Griffioen A et al. Development and evaluation of a hospital discharge information package to empower parents in caring for a child with a fever. BMJ Open. 2018;8:e021697.
36. Thompson AP, Nesari M, Hartling L, Scott SD. Parents' experiences and information needs related to childhood fever: A systematic review. Patient Educ Couns. 2020;103:750-763.
37. NHS Fever. Available from: https://what0-18.nhs.uk/professionals/pharmacists/safety-netting-documents-parents/fever-children-under-5-years.
38. Sinha IP, Brown L, Fulton O et al. Empowering children and young people who have asthma. Arch Dis Child. 2021;106:125-129.
39. Albritton K, Bleyer WA. The management of cancer in the older adolescent. Eur J Cancer. 2003;39:2584-2599.

For peer review only - http://bmjopen.bmj.com/site/about/guidelines.xhtml

40. Borensztajn D, Yeung S, Hagedoorn NN et al. Diversity in the emergency care for febrile children in Europe: a questionnaire study. BMJ Paediatr Open. 2019;3:e000456.
41. Vergouwe Y, Royston P, Moons KG, Altman DG. Development and validation of a prediction model with missing predictor data: a practical approach. J Clin Epidemiol. 2010;63:205-214.
42. Hagedoorn NN, Zachariasse JM, Moll HA. Association between hypotension and serious illness in the emergency department: an observational study. Arch Dis Child. 2020;105:545-551.
43. Stewart JN, McGillivray D, Sussman J, Foster B. The value of routine blood pressure measurement in children presenting to the emergency department with nonurgent problems. J Pediatr. 2008;153:478-483.

For peer review only

For peer review only - http://bmjopen.bmj.com/site/about/guidelines.xhtml

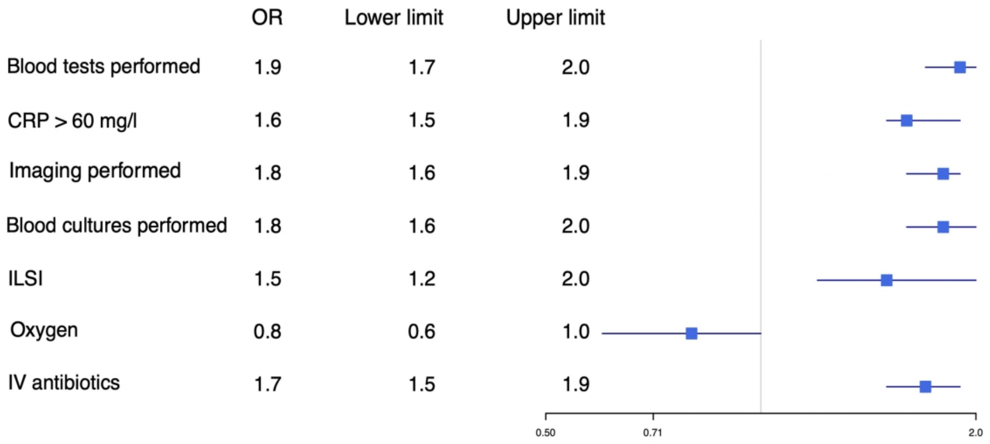

Figure 1

254x118mm (300 x 300 DPI)

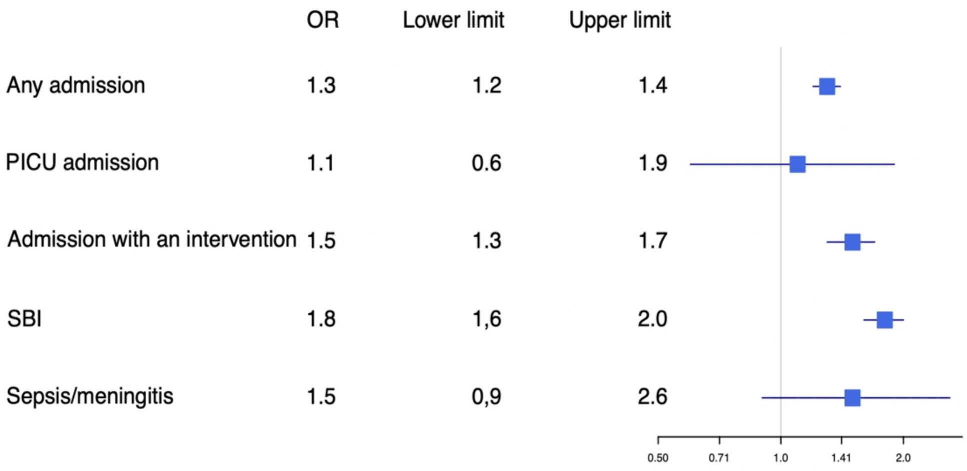

Figure 2

253x127mm (300 x 300 DPI)

	OR	Lower limit	Upper limit
Blood tests performed	1.7	1.5	1.9
CRP > 60 mg/l	1.5	1.3	1.8
Imaging performed	1.6	1.5	1.8
Blood cultures performed	1.5	1.3	1.8
ILSI	1.2	0.8	1.9
Oxygen	0.3	0.2	0.6
IV antibiotics	1.7	1.5	2.0

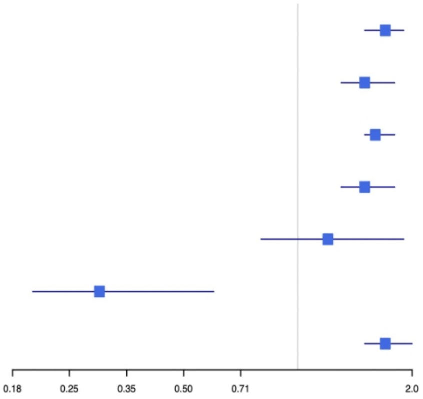

Figure 3

253x114mm (300 x 300 DPI)

	OR	Lower limit	Upper limit
Any admission	1.3	1.1	1.4
PICU admission	1.0	0.3	2.6
Admission with an intervention	1.4	1.2	1.7
SBI	1.8	1,6	2.1
Sepsis/meningitis	2.3	1,1	5.0

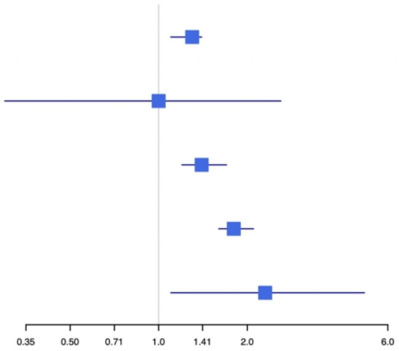

Figure 4

253x101mm (300 x 300 DPI)

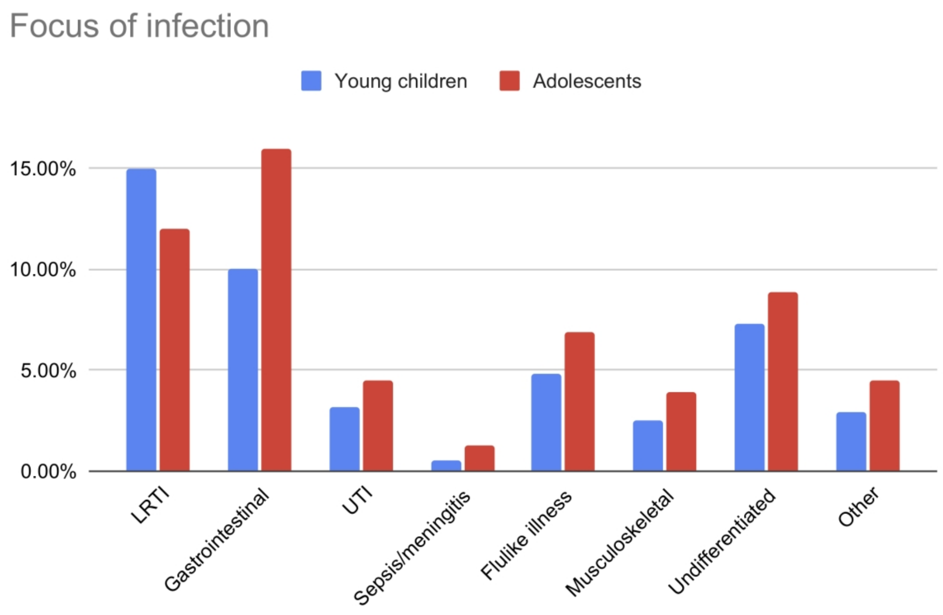

Figure 5

211x153mm (300 x 300 DPI)

PERFORM consortium author list.

PARTNER: IMPERIAL COLLEGE (UK)

Chief investigator/PERFORM coordinator:

Michael Levin

Principal and co-investigators; work package leads (alphabetical order)

Aubrey Cunnington (grant application)

Tisham De (work package lead)

Jethro Herberg (Principle Investigator, Deputy Coordinator, grant application)

Myrsini Kaforou (grant application, work package lead)

Victoria Wright (grant application, Scientific Coordinator)

Research Group (alphabetical order)

Lucas Baumard; Evangelos Bellos; Giselle D'Souza; Rachel Galassini; Dominic Habgood-Coote; Shea Hamilton; Clive Hoggart; Sara Hourmat; Heather Jackson; Ian Maconochie; Stephanie Menikou; Naomi Lin; Samuel Nichols; Ruud Nijman; Ivonne Pena Paz; Priyen Shah; Ching-Fen Shen; Ortensia Vito; Clare Wilson

Clinical recruitment at Imperial College Healthcare NHS Trust (alphabetical order))

Amina Abdulla; Ladan Ali; Sarah Darnell; Rikke Jorgensen; Sobia Mustafa; Salina Persand

Imperial College Faculty of Engineering

Molly Stevens (co-investigator), Eunjung Kim (research group); Benjamin Pierce (research group)

Clinical recruitment at Brighton and Sussex University Hospitals

Katy Fidler (Principle Investigator)

Julia Dudley (Clinical Research Registrar)

Research nurses: Vivien Richmond, Emma Tavliavini

Clinical recruitment at National Cheng Kung University Hospital

Ching-Fen Shen (Principal Investigator); Ching-Chuan Liu (Co-investigator); Shih-Min Wang (Co-investigator), funded by the Center of Clinical Medicine Research, National Cheng Kung University

For peer review only - http://bmjopen.bmj.com/site/about/guidelines.xhtml

SERGAS Partner (Spain)

Principal Investigators

Federico Martinón-Torres[1]

Antonio Salas[1,2]

GENVIP RESEARCH GROUP (in alphabetical order):

Fernando Álvez González[1], Cristina Balo Farto[1], Ruth Barral-Arca[1,2], María Barreiro Castro[1], Xabier Bello[1,2], Mirian Ben García[1], Sandra Carnota[1], Miriam Cebey-López[1], María José Curras-Tuala[1,2], Carlos Durán Suárez[1], Luisa García Vicente[1], Alberto Gómez-Carballa[1,2], Jose Gómez Rial[1], Pilar Leboráns Iglesias[1], Federico Martinón-Torres[1], Nazareth Martinón-Torres[1], José María Martinón Sánchez[1], Belén Mosquera Pérez[1], Jacobo Pardo-Seco[1,2], Lidia Piñeiro Rodríguez[1], Sara Pischedda[1,2], Sara Rey Vázquez[1], Irene Rivero Calle[1], Carmen Rodríguez-Tenreiro[1], Lorenzo Redondo-Collazo[1], Miguel Sadiki Ora[1], Antonio Salas[1,2], Sonia Serén Fernández[1], Cristina Serén Trasorras[1], Marisol Vilas Iglesias[1].

[1] Translational Pediatrics and Infectious Diseases, Pediatrics Department, Hospital Clínico Universitario de Santiago, Santiago de Compostela, Spain, and GENVIP Research Group (www.genvip.org), Instituto de Investigación Sanitaria de Santiago, Universidad de Santiago de Compostela, Galicia, Spain.

[2] Unidade de Xenética, Departamento de Anatomía Patolóxica e Ciencias Forenses, Instituto de Ciencias Forenses, Facultade de Medicina, Universidade de Santiago de Compostela, and GenPop Research Group, Instituto de Investigaciones Sanitarias (IDIS), Hospital Clínico Universitario de Santiago, Galicia, Spain

[3] Fundación Pública Galega de Medicina Xenómica, Servizo Galego de Saúde (SERGAS), Instituto de Investigaciones Sanitarias (IDIS), and Grupo de Medicina Xenómica, Centro de Investigación Biomédica en Red de Enfermedades Raras (CIBERER), Universidade de Santiago de Compostela (USC), Santiago de Compostela, Spain

For peer review only

RSU Partner (Latvia)

Principal Investigator

Dace Zavadska[1,2]

Other RSU group authors (in alphabetical order):

Anda Balode[1,2], Arta Bārzdiņa[1,2], Dārta Deksne[1,2], Dace Gardovska[1,2], Dagne Grāvele[2], Ilze Grope[1,2], Anija Meiere[1,2], Ieva Nokalna[1,2], Jana Pavāre[1,2], Zanda Pučuka[1,2], Katrīna Selecka[1,2], Aleksandra Sidorova[1,2], Dace Svile[2], Urzula Nora Urbāne[1,2].

[1] Riga Stradins university, Riga, Latvia.

[2] Children clinical university hospital, Riga, Latvia.

For peer review only

For peer review only - http://bmjopen.bmj.com/site/about/guidelines.xhtml

Medical Research Council Unit The Gambia (MRCG) at LSHTM Partner

Principal Investigator
Effua Usuf

Additional Investigators
Kalifa Bojang
Syed M. A. Zaman
Fatou Secka
Suzanne Anderson
Anna Rocalsatou Sarr
Momodou Saidykhan
Saffiatou Darboe
Samba Ceesay
Umberto D'alessandro

Medical Research Council Unit The Gambia at LSHTM
P O Box 273,
Fajara, The Gambia

For peer review only

For peer review only - http://bmjopen.bmj.com/site/about/guidelines.xhtml

ERASMUS MC-Sophia Children's Hospital

Principal Investigator

Henriëtte A. Moll[1]

Research group

Dorine M. Borensztajn[1], Nienke N. Hagedoorn, Chantal Tan [1], [1], Clementien L. Vermont[2], Joany Zachariasse [1]

Additional investigator

W Dik [3]

[1] Erasmus MC-Sophia Children's Hospital, Department of General Paediatrics, Rotterdam, the Netherlands

[2] Erasmus MC-Sophia Children's Hospital, Department of Paediatric Infectious Diseases & Immunology, Rotterdam, the Netherlands

[3] Erasmus MC, Department of immunology, Rotterdam, the Netherlands

For peer review only

For peer review only - http://bmjopen.bmj.com/site/about/guidelines.xhtml

Swiss Pediatric Sepsis Study

Principal Investigators*:*

Philipp Agyeman, MD [1] (ORCID 0000-0002-8339-5444), Luregn J Schlapbach, MD, FCICM [2,3] (ORCID 0000-0003-2281-2598)

Clinical recruitment at University Children's Hospital Bern for PERFORM:

Christoph Aebi [1], Verena Wyss [1], Mariama Usman [1]

Principal and co-investigators for the Swiss Pediatric Sepsis Study:

Philipp Agyeman, MD [1], Luregn J Schlapbach, MD, FCICM [2,3], Eric Giannoni, MD [4,5], Martin Stocker, MD [6], Klara M Posfay-Barbe, MD [7], Ulrich Heininger, MD [8], Sara Bernhard-Stirnemann, MD [9], Anita Niederer-Loher, MD [10], Christian Kahlert, MD [10], Giancarlo Natalucci, MD [11], Christa Relly, MD [12], Thomas Riedel, MD [13], Christoph Aebi, MD [1], Christoph Berger, MD [12] **for the Swiss Pediatric Sepsis Study**

Affiliations:

[1] Department of Pediatrics, Inselspital, Bern University Hospital, University of Bern, Switzerland

[2] Neonatal and Pediatric Intensive Care Unit, Children's Research Center, University Children's Hospital Zurich, University of Zurich, Zurich, Switzerland

[3]Child Health Research Centre, University of Queensland, and Queensland Children`s Hospital, Brisbane, Australia

[4] Clinic of Neonatology, Department Mother-Woman-Child, Lausanne University Hospital and University of Lausanne, Switzerland

[5] Infectious Diseases Service, Department of Medicine, Lausanne University Hospital and University of Lausanne, Switzerland

[6] Department of Pediatrics, Children's Hospital Lucerne, Lucerne, Switzerland

[7] Pediatric Infectious Diseases Unit, Children's Hospital of Geneva, University Hospitals of Geneva, Geneva, Switzerland

[8] Infectious Diseases and Vaccinology, University of Basel Children's Hospital, Basel, Switzerland

[9] Children's Hospital Aarau, Aarau, Switzerland

[10] Division of Infectious Diseases and Hospital Epidemiology, Children's Hospital of Eastern Switzerland St. Gallen, St. Gallen, Switzerland

[11] Department of Neonatology, University Hospital Zurich, Zurich, Switzerland

[12] Division of Infectious Diseases and Hospital Epidemiology, and Children's Research Center, University Children's Hospital Zurich, Switzerland

[13] Children's Hospital Chur, Chur, Switzerland

For peer review only - http://bmjopen.bmj.com/site/about/guidelines.xhtml

Liverpool Partner

Principal Investigators

Enitan D Carrol[1,2,3]

Stéphane Paulus [1,]

Research Group (in alphabetical order):

Elizabeth Cocklin[1], Rebecca Jennings[4], Joanne Johnston[4], Simon Leigh[1], Karen Newall[4], Sam Romaine[1]

[1] Department of Clinical Infection, Microbiology and Immunology, University of Liverpool Institute of Infection and Global Health , Liverpool, England

[2] Alder Hey Children's Hospital, Department of Infectious Diseases, Eaton Road, Liverpool, L12 2AP

[3] Liverpool Health Partners, 1st Floor, Liverpool Science Park, 131 Mount Pleasant, Liverpool, L3 5TF

[4]Alder Hey Children's Hospital, Clinical Research Business Unit, Eaton Road, Liverpool, L12 2AP

For peer review only

For peer review only - http://bmjopen.bmj.com/site/about/guidelines.xhtml

NKUA Partner (Greece)

Principal investigator: Professor **Maria Tsolia** (all activities)

Investigator/Research fellow: **Irini Eleftheriou** (all activities)

Additional investigators:

Recruitment: Maria Tambouratzi

Lab: Antonis Marmarinos (Quality Manager)

Lab: Marietta Xagorari

Kelly Syggelou

2nd Department of Pediatrics, National and Kapodistrian University of Athens,

"P. and A. Kyriakou" Children's Hospital

Thivon and Levadias

Goudi, Athens

For peer review only

For peer review only - http://bmjopen.bmj.com/site/about/guidelines.xhtml

Micropathology Ltd :

Principal Investigator:

Professor Colin Fink[1], Clinical Microbiologist

Additional investigators

Dr Marie Voice[1], Post doc scientist

Dr. Leo Calvo-Bado[1], Post doc scientist

[1] Micropathology Ltd, The Venture Center, University of Warwick Science Park, Sir William Lyons Road, Coventry, CV4 7EZ.

For peer review only

For peer review only - http://bmjopen.bmj.com/site/about/guidelines.xhtml

Medical University of Graz, Austria (MUG)

Principal Investigator:

Werner Zenz[1] (all activities)

Co-investigators (in alphabetical order)

Benno Kohlmaier[1] (all activities)

Nina A. Schweintzger[1] (all activities)

Manfred G. Sagmeister[1] (study design, consortium wide sample management)

Research team

Daniela S. Kohlfürst[1] (study design)

Christoph Zurl[1] (BIVA PIC)

Alexander Binder[1] (grant application)

Recruitment team, data managers, (in alphabetical order):

Susanne Hösele[1], Manuel Leitner[1], Lena Pölz[1], Glorija Rajic[1],

Clinical recruitment partners (in alphabetical order):

Sebastian Bauchinger[1], Hinrich Baumgart[4], Martin Benesch[3], Astrid Ceolotto[1], Ernst Eber[2], Siegfried Gallistl[1], Gunther Gores[5], Harald Haidl[1], Almuthe Hauer[1], Christa Hude[1], Markus Keldorfer[5], Larissa Krenn[4], Heidemarie Pilch[5], Andreas Pfleger[2], Klaus Pfurtscheller[4], Gudrun Nordberg[5], Tobias Niedrist[8], Siegfried Rödl[4], Andrea Skrabl-Baumgartner[1], Matthias Sperl[7], Laura Stampfer[5], Volker Strenger[3], Holger Till[6], Andreas Trobisch[5], Sabine Löffler[5]

Author Affiliations:

[1] Department of Pediatrics and Adolescent Medicine, Division of General Pediatrics, Medical University of Graz, Graz, Austria

[2]Department of Pediatric Pulmonology, Medical University of Graz, Graz, Austria

[3]Department of Pediatric Hematooncoloy, Medical University of Graz, Graz, Austria

[4]Paediatric Intensive Care Unit, Medical University of Graz, Graz, Austria

For peer review only - http://bmjopen.bmj.com/site/about/guidelines.xhtml

[5]University Clinic of Paediatrics and Adolescent Medicine Graz, Medical University Graz, Graz,Austria

[6]Department of Paediatric and Adolescence Surgery, Medical University Graz, Graz, Austria

[7]Department of Pediatric Orthopedics, Medical University Graz, Graz, Austria

[8]Clinical Institute of Medical and Chemical Laboratory Diagnostics, Medical University Graz, Graz, Austria

For peer review only

For peer review only - http://bmjopen.bmj.com/site/about/guidelines.xhtml

London School of Hygiene and Tropical Medicine

WP 1 WP2, WP5

Principal Investigator:

Dr Shunmay Yeung[1,2 3] PhD, MBBS, FRCPCH, MRCP, DTM&H

Research Group

Dr Juan Emmanuel Dewez[1] MD, DTM&H, MSc

Prof Martin Hibberd [1] BSc, PhD

Mr David Bath[2] MSc, MAppFin, BA(Hons)

Dr Alec Miners[2] BA(Hons), MSc, PhD

Dr Ruud Nijman[3] PhD MSc MD MRCPCH

Dr Catherine Wedderburn[1] BA, MBChB, DTM&H, MSc, MRCPCH

Ms Anne Meierford[1] MSc, BMedSc, BMBS

Dr Baptiste Leurent[4], PhD, MSc

1. Faculty of Infectious and Tropical Disease, London School of Hygiene and Tropical Medicine, London, UK
2. Faculty of Public Health and Policy, London School of Hygiene and Tropical Medicine, London, UK
3. Department of Paediatrics, St. Mary's Hospital Imperial College Hospital, London, UK
4. Faculty of Epidemiology and Population Health, London School of Hygiene and Tropical Medicine, London, UK

For peer review only

Radboud University Medical Center (RUMC), The Netherlands

Principal Investigators:

Ronald de Groot [1], Michiel van der Flier [1,2,3], Marien I. de Jonge[1]

Co-investigators Radboud University Medical Center (in alphabetical order):

Koen van Aerde[1,2], Wynand Alkema[1], Bryan van den Broek[1], Jolein Gloerich[1], Alain J. van Gool[1], Stefanie Henriet[1,2], Martijn Huijnen[1], Ria Philipsen[1], Esther Willems[1]

Investigators PeDBIG PERFORM DUTCH CLINICAL NETWORK (in alphabetical order):

G.P.J.M. Gerrits[8], M. van Leur[8,] J. Heidema [4],L. de Haan[1,2] C.J. Miedema [5], C. Neeleman [1] C.C. Obihara [6], G.A. Tramper-Stranders7[6]

1. Radboud University Medical Center, Nijmegen, The Netherlands
2. Amalia Children's Hospital, Nijmegen, The Netherlands
3. Wilhelmina Children's Hospital, University Medical Center Utrecht, Utrecht, The Netherlands
4. St. Antonius Hospital, Nieuwegein, The Netherlands
5. Catharina Hospital, Eindhoven, The Netherlands
6. ETZ Elisabeth, Tilburg, The Netherlands
7. Franciscus Gasthuis, Rotterdam, The Netherlands
8. Canisius Wilhelmina Hospital, Nijmegen, The Netherlands

For peer review only

Oxford team (UK)

Principal Investigators

Andrew J. Pollard[1,2], Rama Kandasamy[1,2], Stéphane Paulus [1,2]

Additional Investigators

Michael J. Carter[1,2], Daniel O'Connor[1,2], Sagida Bibi[1,2], Dominic F. Kelly[1,2], Meeru Gurung[3], Stephen Thorson[3], Imran Ansari[3], David R. Murdoch[4], Shrijana Shrestha[3], Zoe Oliver[5]

Author Affiliations:

[1]Oxford Vaccine Group, Department of Paediatrics, University of Oxford, Oxford, United Kingdom.

[2]NIHR Oxford Biomedical Research Centre, Oxford, United Kingdom.

[3]Paediatric Research Unit, Patan Academy of Health Sciences, Kathmandu, Nepal.

[4]Department of Pathology, University of Otago, Christchurch, New Zealand.

[5] Department of Paediatrics, University of Oxford.

For peer review only

For peer review only - http://bmjopen.bmj.com/site/about/guidelines.xhtml

Newcastle University, Newcastle upon Tyne, (UK)

Principal Investigator:

Marieke Emonts [1,2,3] (all activities)

Co-investigators

Emma Lim[2,3,7] (all activities)

Lucille Valentine[4]

Recruitment team (alphabetical), data-managers, and GNCH Research unit:

Karen Allen[5], Kathryn Bell[5], Adora Chan[5], Stephen Crulley[5], Kirsty Devine[5], Daniel Fabian[5], Sharon King[5], Paul McAlinden[5], Sam McDonald[5], Anne McDonnell2,[5], Ailsa Pickering[2,5], Evelyn Thomson[5], Amanda Wood[5], Diane Wallia[5], Phil Woodsford[5],

Sample processing: Frances Baxter[5], Ashley Bell[5], Mathew Rhodes[5]

PICU recruitment

Rachel Agbeko[8]

Christine Mackerness[8]

Students MOFICHE

Bryan Baas[2], Lieke Kloosterhuis[2], Wilma Oosthoek[2]

Students/medical staff PERFORM

Tasnim Arif[6], Joshua Bennet[2], Kalvin Collings[2], Ilona van der Giessen[2], Alex Martin[2], Aqeela Rashid[6], Emily Rowlands[2], Gabriella de Vries[2], Fabian van der Velden[2]

Engagement work/ethics/cost effectiveness

Lucille Valentine [4], Mike Martin[9], Ravi Mistry[2], Lucille Valentine[4]

Author Affiliations:

[1] Translational and Clinical Research Institute, Newcastle University, Newcastle upon Tyne UK

[2]Great North Children's Hospital, Paediatric Immunology, Infectious Diseases & Allergy, Newcastle upon Tyne Hospitals NHS Foundation Trust, Newcastle upon Tyne, United Kingdom.

For peer review only - http://bmjopen.bmj.com/site/about/guidelines.xhtml

[3]NIHR Newcastle Biomedical Research Centre based at Newcastle upon Tyne Hospitals NHS Trust and Newcastle University, Westgate Rd, Newcastle upon Tyne NE4 5PL, United Kingdom

[4]Newcastle University Business School, Centre for Knowledge, Innovation, Technology and Enterprise (KITE), Newcastle upon Tyne, United Kingdom

[5]Great North Children's Hospital, Research Unit, Newcastle upon Tyne Hospitals NHS Foundation Trust, Newcastle upon Tyne, United Kingdom.

[6]Great North Children's Hospital, Paediatric Oncology, Newcastle upon Tyne Hospitals NHS Foundation Trust, Newcastle upon Tyne, United Kingdom.

[7]Population Health Sciences Institute, Newcastle University, Newcastle upon Tyne, UK

[8]Great North Children's Hospital, Paediatric Intensive Care Unit, Newcastle upon Tyne Hospitals NHS Foundation Trust, Newcastle upon Tyne, United Kingdom.

[9]Northumbria University, Newcastle upon Tyne, United Kingdom.

For peer review only

LMU Munich Partner (Germany)

Principal Investigator:

Ulrich von Both[1,2] MD, FRCPCH (all activities)

Research group:

Laura Kolberg[1] MSc (all activities)

Manuela Zwerenz[1] MSc, Judith Buschbeck[1] PhD

Clinical recruitment partners (in alphabetical order):

Christoph Bidlingmaier[3], Vera Binder[4], Katharina Danhauser[5], Nikolaus Haas[10], Matthias Griese[6], Tobias Feuchtinger[4], Julia Keil[9], Matthias Kappler[6], Eberhard Lurz[7], Georg Muench[8], Karl Reiter[9], Carola Schoen[9]

Author Affiliations:

[1]Div. Paediatric Infectious Diseases, Hauner Children's Hospital, University Hospital, Ludwig Maximilians University (LMU), Munich, Germany

[2]German Center for Infection Research (DZIF), Partner Site Munich, Munich, Germany

[3]Div. of General Paediatrics, [4]Div. Paediatric Haematology & Oncology, [5]Div. of Paediatric Rheumatology, [6]Div. of Paediatric Pulmonology, [7]Div. of Paediatric Gastroenterology, [8]Neonatal Intensive Care Unit, [9]Paediatric Intensive Care Unit Hauner Children's Hospital, University Hospital, Ludwig Maximilians University (LMU), Munich, Germany, [10]Department Pediatric Cardiology and Pediatric Intensive Care, University Hospital, Ludwig Maximilians University (LMU), Munich, Germany

For peer review only

For peer review only - http://bmjopen.bmj.com/site/about/guidelines.xhtml

bioMérieux, France

Principal Investigator:
François Mallet[1,2, 3]

Research Group:
Karen Brengel-Pesce[1,2, 3]
Alexandre Pachot[1]
Marine Mommert[1,2]

[1]Open Innovation & Partnerships (OIP), bioMérieux S.A., Marcy l'Etoile, France
[2] Joint research unit Hospice Civils de Lyon - bioMérieux, Centre Hospitalier Lyon Sud, 165 Chemin du Grand Revoyet, 69310 Pierre-Bénite, France
[3] EA 7426 Pathophysiology of Injury-induced Immunosuppression, University of Lyon1-Hospices Civils de Lyon-bioMérieux, Hôpital Edouard Herriot, 5 Place d'Arsonval, 69437 Lyon Cedex 3, France

Department of Infectious Diseases, University Medical Centre Ljubljana, Slovenia

Principal Investigator:

Marko Pokorn[1,2,3] MD, PhD

Research Group:

Mojca Kolnik[1] MD, Katarina Vincek[1] MD, Tina Plankar Srovin[1] MD, PhD, Natalija Bahovec[1] MD, Petra Prunk[1] MD, Veronika Osterman[1] MD, Tanja Avramoska[1] MD

Affiliations:

[1]Department of Infectious Diseases, University Medical Centre Ljubljana, Japljeva 2, SI-1525 Ljubljana, Slovenia

[2]University Childrens' Hospital, University Medical Centre Ljubljana, Ljubljana, Slovenia

[3]Department of Infectious Diseases and Epidemiology, Faculty of Medicine, University of Ljubljana, Slovenia

For peer review only - http://bmjopen.bmj.com/site/about/guidelines.xhtml

Amsterdam, Academic Medical Hospital & Sanquin Research Institute (NL)

Principal Investigator:

Taco Kuijpers [1,2]

Co-investigators

Ilse Jongerius [2]

Recruitment team (EUCLIDS, PERFORM):

J.M. van den Berg[1], D. Schonenberg[1], A.M. Barendregt[1], D. Pajkrt[1], M. van der Kuip[1,3], A.M. van Furth[1,3]

Students PERFORM

Evelien Sprenkeler [2], Judith Zandstra [2],

Technical support PERFORM

G. van Mierlo [2], J. Geissler [2]

Author Affiliations:

[1] Amsterdam University Medical Center (Amsterdam UMC), location Academic Medical Center (AMC), Dept of Pediatric Immunology, Rheumatology and Infectious Diseases, University of Amsterdam, Amsterdam, the Netherlands

[2] Sanquin Research Institute, & Landsteiner Laboratory at the AMC, University of Amsterdam, Amsterdam, the Netherlands.

[3] Amsterdam University Medical Center (Amsterdam UMC), location Vrije Universiteit Medical Center (VUMC), Dept of Pediatric Infectious Diseases and Immunology, Free University (VU), Amsterdam, the Netherlands (former affiliation)

For peer review only - http://bmjopen.bmj.com/site/about/guidelines.xhtml

Appendix 2: Participating EDs

Hospital	Country, city	Hospital type	Total annual paediatric ED visits	Total period of inclusion	Period of inclusion per month	Number of patients included
Medizinische Universität Graz	Austria, Graz	University	10,000-30,000	1-1-2017 – 31-12-2018	10 days	2243
Dr. von Hauner Children's Hospital	Germany, Munich	Teaching	10,000-30,000	1-1-2017 – 31-12-2018	1 week	1175
P. and A. Kyriakou Children's Hospital	Greece, Athens	University	>30,000	1-1-2017 – 1-5-2018	1-2 weeks	4549
Children clinical university hospital	Latvia, Riga	Teaching	>30,000	1-1-2017 – 31-12-2018	All	9000
Univerzitetni Klinični Center	Slovenia, Ljubljana	University	<10,000	1-1-2017 – 31-12-2018	All	3659
Hospital Clínico Universitario	Spain, Santiago de Compostela	University	>30,000	1-1-2017 – 1-5-2018	1-2 weeks	3877
Erasmus MC-Sophia Children's Hospital	The Netherlands, Rotterdam	University	<10,000	1-1-2017 – 1-4-2018	All	1681
RadboudUMC	The Netherlands, Nijmegen	University	<10,000	1-1-2017 – 1-4-2018	All	676
Canisius Wilhelmina Ziekenhuis	The Netherlands, Nijmegen	Teaching	<10,000	1-1-2017 – 31-12-2018	2 weeks	415
Alder Hey Children's Hospital	United Kingdom, Liverpool	Teaching	>30,000	1-1-2017 – 31-12-2018	1 week	1624
St. Mary's Hospital	United Kingdom, London	University	10,000-30,000	1-1-2017 – 31-12-2018	All	5714
Great North Children's Hospital	United Kingdom, Newcastle upon Tyne	University	>30,000	1-4-2017 – 1-4-2018	2 weeks	3870

For peer review only

Appendix 3. Immediate life-saving interventions

1. Airway and breathing support, including intubation or emergent noninvasive positive pressure ventilation.
2. Electrical therapy, including defibrillation, emergent cardioversion, or external pacing.
3. Procedures, including chest needle decompression, pericardiocentesis, or open thoracotomy.
4. Hemodynamic support, including significant intravenous fluid resuscitation in the setting of hypotension, blood administration, or control of major bleeding.
5. Emergency medications, including naloxone, dextrose, atropine, adenosine, epinephrine, or vasopressors

For peer review only

For peer review only - http://bmjopen.bmj.com/site/about/guidelines.xhtml

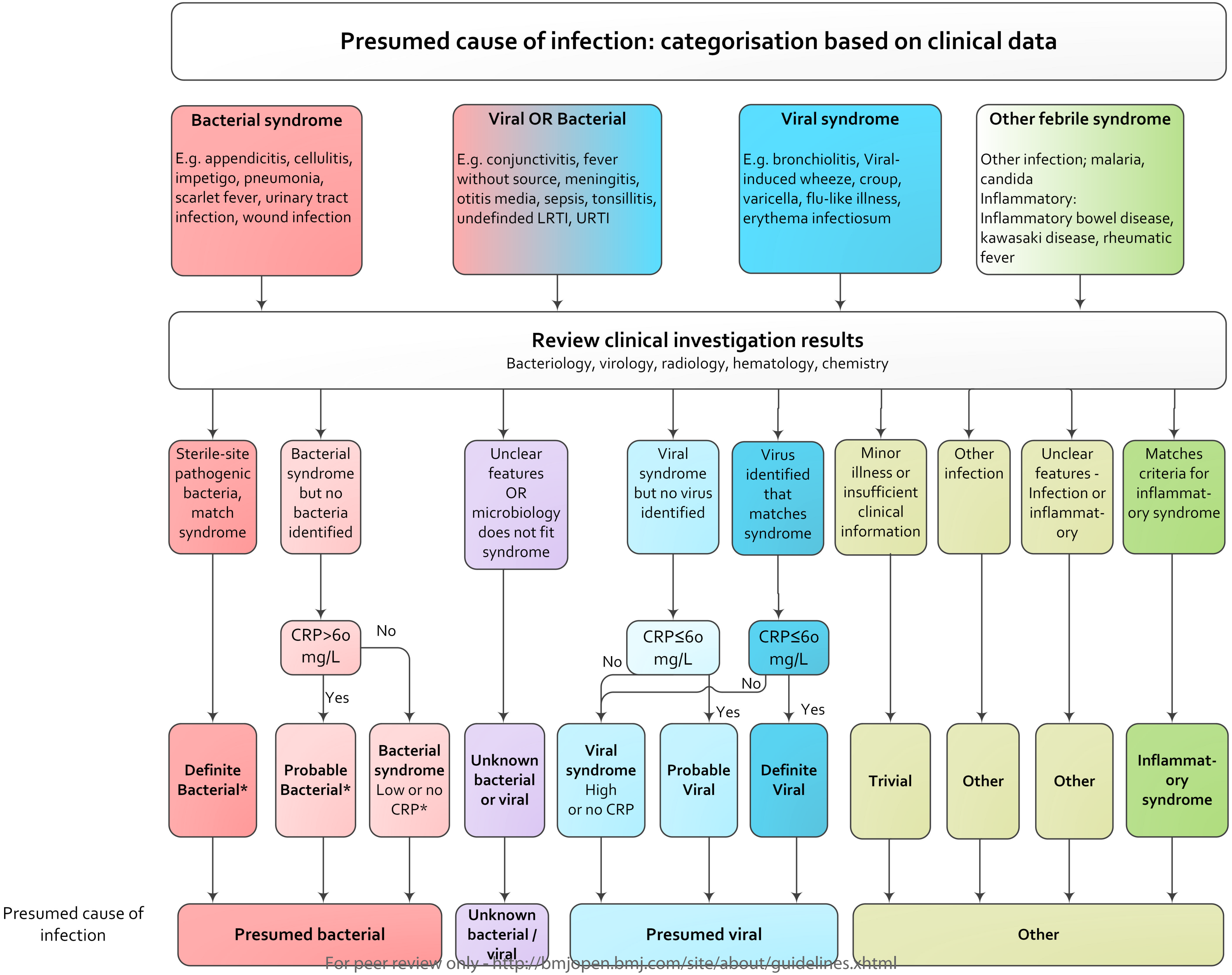
Presumed cause of infection: categorisation based on clinical data
Bacterial syndrome
E.g. appendicitis, cellulitis, impetigo, pneumonia, scarlet fever, urinary tract infection, wound infection
Viral OR Bacterial
E.g. conjunctivitis, fever without source, meningitis, otitis media, sepsis, tonsillitis, undefinded LRTI, URTI
Viral syndrome
E.g. bronchiolitis, Viral-induced wheeze, croup, varicella, flu-like illness, erythema infectiosum
Other febrile syndrome
Other infection; malaria, candida
Inflammatory:
Inflammatory bowel disease, kawasaki disease, rheumatic fever
Review clinical investigation results
Bacteriology, virology, radiology, hematology, chemistry
Sterile-site pathogenic bacteria, match syndrome
Bacterial syndrome but no bacteria identified
Unclear features OR microbiology does not fit syndrome
Viral syndrome but no virus identified
Virus identified that matches syndrome
Minor illness or insufficient clinical information
Other infection
Unclear features - Infection or inflammatory
Matches criteria for inflammatory syndrome
CRP>60 mg/L
No
Yes
CRP≤60 mg/L
No
Yes
CRP≤60 mg/L
No
Yes
Definite Bacterial*
Probable Bacterial*
Bacterial syndrome Low or no CRP*
Unknown bacterial or viral
Viral syndrome High or no CRP
Probable Viral
Definite Viral
Trivial
Other
Other
Inflammatory syndrome
Presumed cause of infection
Presumed bacterial
Unknown bacterial / viral
Presumed viral
Other

Appendix 5: Comorbidity.

	Children 3 months- 12 years N = 34,843 N (%)	Children > 12 years N = 2,577 N (%)
No comorbidity	28,881 (83·7)	1,833 (71·5)
Non-complex comorbidity	4,302 (12·5)	489 (19·1)
Complex comorbidity	1,332 (3·9)	241 (9·4)
Type of comorbidity		
Neurological & psychomotor delay	1,604 (28·5)	298 (40·8)
Pulmonary	1,224 (21·7)	182 (24·9)
Prematurity	945 (16·8)	20 (2·7)
Urological/nephrological	634 (11·3)	57 (7·8)
Malignancy & immunodeficiency	583 (10·4)	162 (22·2)
Cardiac	557 (9·9)	58 (7·9)
Gastro-intestinal	422 (7·5)	81 (11·1)
Musculoskeletal	171 (3·0)	55 (7·5)
Metabolic	165 (2·9)	53 (7·3)
Endocrine	63 (1·1)	27 (3·7)
Other comorbidity	729 (12·9)	83 (11·3)

Type of comorbidity displayed as percentage of children with comorbidity.

For peer review only

Appendix 6: Differences in patient characteristics between young children and adolescents with a final diagnosis of SBI. [a]

	OR	Lower limit	Upper limit
General patient characteristics			
Triage urgency high	1.0	0.8	1.3
Referral by EMS	1.0	1.0	1.1
Comorbidity	1.7	1.4	2.2
Vital signs			
Tachycardia	0.9	0.7	1.2
Tachypnoea	0.5	0.3	0.6
Prolonged capillary refill	1.1	0.6	2.2

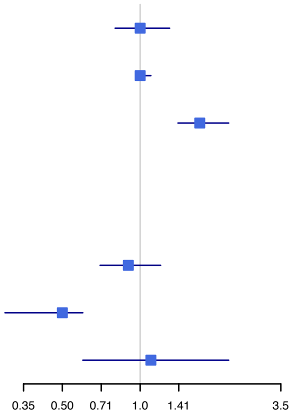

	OR	Lower limit	Upper limit
NICE fever guideline alarming signs			
Ill appearance	1.4	1.1	1.8
Non−blanching rash	0.8	0.3	2.1
Blanching rash	0.7	0.4	1.1
Increased work of breathing	0.4	0.3	0.7
Neurological signs	1.1	0.3	3.1
NICE sepsis guideline high risk criteria			
1 or more	0.4	0.3	0.5
2 or more	0.3	0.2	0.4
3 or more	0.2	0.1	0.3

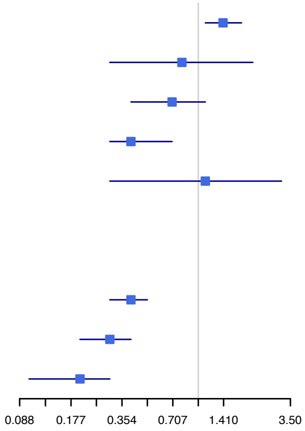

[a] Younger children used as reference. Adjusted for hospital, sex, duration of fever, previous medical care, time of arrival and comorbidity. * According to APLS cut-off values by age.

For peer review only

STROBE Statement—Checklist of items that should be included in reports of ***cohort studies***

	Item No	Recommendation	Page No
**5Title and abstract**	1	(*a*) Indicate the study's design with a commonly used term in the title or the abstract	5
		(*b*) Provide in the abstract an informative and balanced summary of what was done and what was found	5
**Introduction**			
Background/rationale	2	Explain the scientific background and rationale for the investigation being reported	7
Objectives	3	State specific objectives, including any prespecified hypotheses	7
**Methods**			
Study design	4	Present key elements of study design early in the paper	8
Setting	5	Describe the setting, locations, and relevant dates, including periods of recruitment, exposure, follow-up, and data collection	8
Participants	6	(*a*) Give the eligibility criteria, and the sources and methods of selection of participants. Describe methods of follow-up	8
		(*b*) For matched studies, give matching criteria and number of exposed and unexposed	
Variables	7	Clearly define all outcomes, exposures, predictors, potential confounders, and effect modifiers. Give diagnostic criteria, if applicable	9,10
Data sources/ measurement	8*	For each variable of interest, give sources of data and details of methods of assessment (measurement). Describe comparability of assessment methods if there is more than one group	9,10
Bias	9	Describe any efforts to address potential sources of bias	11
Study size	10	Explain how the study size was arrived at	9
Quantitative variables	11	Explain how quantitative variables were handled in the analyses. If applicable, describe which groupings were chosen and why	9,10
Statistical methods	12	(*a*) Describe all statistical methods, including those used to control for confounding	11,12
		(*b*) Describe any methods used to examine subgroups and interactions	
		(*c*) Explain how missing data were addressed	
		(*d*) If applicable, explain how loss to follow-up was addressed	
		(*e*) Describe any sensitivity analyses	
**Results**			
Participants	13*	(a) Report numbers of individuals at each stage of study—eg numbers potentially eligible, examined for eligibility, confirmed eligible, included in the study, completing follow-up, and analysed	12
		(b) Give reasons for non-participation at each stage	
		(c) Consider use of a flow diagram	
Descriptive data	14*	(a) Give characteristics of study participants (eg demographic, clinical, social) and information on exposures and potential confounders	12,13, 24
		(b) Indicate number of participants with missing data for each variable of interest	
		(c) Summarise follow-up time (eg, average and total amount)	
Outcome data	15*	Report numbers of outcome events or summary measures over time	12,13

For peer review only - http://bmjopen.bmj.com/site/about/guidelines.xhtml

Main results	16	(*a*) Give unadjusted estimates and, if applicable, confounder-adjusted estimates and their precision (eg, 95% confidence interval). Make clear which confounders were adjusted for and why they were included<br>(*b*) Report category boundaries when continuous variables were categorized<br>(*c*) If relevant, consider translating estimates of relative risk into absolute risk for a meaningful time period	11-13<br>27
Other analyses	17	Report other analyses done—eg analyses of subgroups and interactions, and sensitivity analyses	13,14
**Discussion**			
Key results	18	Summarise key results with reference to study objectives	14,15
Limitations	19	Discuss limitations of the study, taking into account sources of potential bias or imprecision. Discuss both direction and magnitude of any potential bias	16,17
Interpretation	20	Give a cautious overall interpretation of results considering objectives, limitations, multiplicity of analyses, results from similar studies, and other relevant evidence	14-17
Generalisability	21	Discuss the generalisability (external validity) of the study results	16,17
**Other information**			
Funding	22	Give the source of funding and the role of the funders for the present study and, if applicable, for the original study on which the present article is based	Author statement form

*Give information separately for exposed and unexposed groups.

Note: An Explanation and Elaboration article discusses each checklist item and gives methodological background and published examples of transparent reporting. The STROBE checklist is best used in conjunction with this article (freely available on the Web sites of PLoS Medicine at http://www.plosmedicine.org/, Annals of Internal Medicine at http://www.annals.org/, and Epidemiology at http://www.epidem.com/). Information on the STROBE Initiative is available at http://www.strobe-statement.org.

For peer review only
